# A taxonomic review of the *Neoserica* (sensu lato) *abnormis* group (Coleoptera, Scarabaeidae, Sericini)

**DOI:** 10.3897/zookeys.439.8055

**Published:** 2014-09-08

**Authors:** Dirk Ahrens, Wan-Gang Liu, Silvia Fabrizi, Ming Bai, Xing-Ke Yang

**Affiliations:** 1 Key Laboratory of Zoological Systematics and Evolution, Institute of Zoology, Chinese Academy of Sciences, Box 92, No. 1, Beichen West Road, Chaoyang District, Beijing, 100101, P.R. China; 2 Zoologisches Forschungsmuseum A. Koenig, Adenauerallee 160, 53113 Bonn, Germany

**Keywords:** Beetles, chafers, *Neoserica*, Indochina, China, new species

## Abstract

The present paper revises the species belonging to the *Neoserica* (sensu lato) *abnormis* group, so far known only with two nominal species. Twenty new species are herein described from Indochina and southern China: *N. abnormoides* **sp. n.** (Vietnam, China), *N. allolaotica* **sp. n.**, *N. namthaensis* **sp. n.**, *N. simplicissima* **sp. n.** (Laos), *N. thailandensis* **sp. n.** (Thailand), *N. alloputaoana* **sp. n.**, *N. kanphantensis* **sp. n.**, *N. natmatoungensis* **sp. n.**, *N. putaoana* **sp. n.**, *N. taunggyiana* **sp. n.** (Myanmar), *N. lamellosa* **sp. n.**, *N. tonkinea* **sp. n.** (Vietnam), *N. bairailingshanica* **sp. n.**, *N. euyunnanica* **sp. n.**, *N. huangi* **sp. n.**, *N. jiangxiensis* **sp. n.**, *N. trifida* **sp. n.**, *N. yaoi* **sp. n.**, *N. yingjiangensis* **sp. n.** (China), *N. cardamomensis* **sp. n.** (Indochina and southern China). One new combination is established: *Neoserica ponderosa* Arrow, 1946, comb. n. The lectotypes of *Neoserica abnormis* Moser, 1908 and the taxonomically uncertain *N. inclinata* Brenske, 1898, which very likely also belongs to this species group, are designated herein. A key to the species and to species groups is given, the genitalia of all species including their habitus are illustrated. Maps of species distribution are included.

## Introduction

*Neoserica* Brenske, 1897 comprises ca. 200 taxa and is one of the most species-rich groups of Sericini. Since the revision of the type species (Pope 1960) and the redefinition of the genus (Ahrens 2003), many other species so far grouped under *Neoserica* and being not directly related to *Neoserica* sensu stricto (Ahrens 2003). They are currently grouped preliminarily as *Neoserica* sensu lato (e.g. Ahrens 2004), a collective group that was found to be not monophyletic (Ahrens and Vogler 2008) and being neither related to *Neoserica* sensu stricto (Ahrens 2003). This paper is part of a series of taxonomic revisions (Ahrens et al. 2014, Ahrens et al. in press) based on which hopefully their relationship and their right classification can be subsequently established.

In the current study, we investigated all the taxa closely related to *Neoserica abnormis* Moser, 1908, which is among the largest species of Sericine chafers worldwide, with a body size up to 17 mm. According to our present knowledge, the species group is distributed in southern China and Indochina being mainly restricted to the higher elevated regions. While the *Neoserica abnormis* group was identified as the sister of *Chrysoserica* Brenske, 1897 by a morphology-based phylogenetic analysis (Ahrens 2012), a molecular phylogeny (Ahrens and Vogler 2008) placed it (with a single included species) as sister of *Nepaloserica* Frey, 1965.

## Material and methods

Terms and methods used for measurements, specimen dissection and preparation of genitalia are the same as used by Ahrens (2004). Data from specimens examined are cited in the text with original label contents given in quotation marks, multiple labels are separated by a “/”. Measurements refer to the maximum extension of the specimen or the named structure. Male genitalia were glued to a small pointed card and photographed in both lateral and dorsal views using a stereomicroscope Leica M125 with a Leica DC420C digital camera. In the automontage software as implemented in Leica Application Suite (V3.3.0), a number of single focused images were combined in order to obtain an entirely focused image. The resulting images were subsequently digitally edited edited to eliminate the background. The distribution maps were generated using Q-GIS 2.0.1 and Adobe Photoshop CS4 software. The key to species groups of *Neoserica* (sensu lato) provided here is currently suitable only for the series of specimens containing both sexes.

Abbreviations used in the text for collection depositories are as follows:

BMNH The Natural History Museum, London, UK;

CPPB Collection P. Pacholátko, Brno, Czech Republic;

CNAR Collection A. Napolov, Riga, Latvia;

HBUM Museum of Hebei University, Baoding (Hebei Prov.), China;

IZAS Institute of Zoology, Chinese Academy of Sciences, Beijing, China;

MNHN Museum national d’Histoire naturelle, Paris, France;

NHMB Naturhistorisches Museum, Basel, Switzerland;

NHRS Naturhistoriska Riksmuseet Stockholm, Sweden;

NKUT Nankai University, Tianjin, China;

NMPC National Museum Prague (Natural History), Czech Republic;

NUYS Northwest A & F University, Yangling (Shaanxi Prov.), China.

SMTD Staatliches Museum für Tierkunde, Dresden; Germany.;

ZFMK Zoologisches Forschungsinstitut und Museum A. Koenig, Bonn; Germany;

ZMHB Zoologisches Museum der Humboldt-Universität, Berlin; Germany;

ZSMC Zoologische Staatssammlung, München; Germany.

## Results

### Key to species groups of *Neoserica* (sensu lato)

**Table:** 

1	Hypomeron not carinate	*Tetraserica* Ahrens, 2004
1’	Hypomeron carinate	2
2	Antennal club in female composed of 3 antennomeres	*Neoserica vulpes* group, *Neoserica lubrica* group, *Neoserica pilosula* group, *Neoserica calva* group, *Anomalophylla* Reitter, 1887, *Gynaecoserica* Brenske, 1896, *Leuroserica* Arrow, 1946, *Sericania* Motschulsky, 1860, *Calloserica* Brenske, 1894, *Lasioserica* Brenske, 1896, *Gastroserica* Brenske, 1897, *Neoserica* (s.str.) Brenske, 1894, *Trioserica* Moser, 1922, *Microserica* Brenske, 1894, *Oxyserica* Brenske, 1900, other *Neoserica* (s. l.)
2’	Antennal club in female composed of more than 3 antennomeres	3
3	Metatibia slender and long	4
3’	Metatibia shorter and wide	Neoserica (s. l.) uniformis group & *Neoserica multifoliata* group (from Indochina)
4	Antennal club of males with 7 antennomeres	5
4’	Antennal club of males with 7, 6 or less antennomeres	6
5	Metafemur with a continuously serrated line adjacent to its anterior margin. Protibia more or less distinctly tridentate	*Neoserica septemlamellata* group
5’	Metafemur without a continuously serrated line adjacent to the anterior margin. Protibia always distinctly bidentate	*Nepaloserica* Frey, 1965
6	Base of labroclypeus dull. Antennal club of males with 6 antennomeres	7
6’	Antennal club of males with 5 or 4 antennomeres	8
7	Angle between base of hypomeron and that of pronotum strongly rounded, angle of surfaces of hypomeron and pronotum basally blunt. Hypomeron basally strongly produced ventrally and transversely sulcate	*Lepidoserica* Nikolaev, 1979
7’	Angle between base of hypomeron and that of pronotum sharp, angle between surfaces of hypomeron and pronotum sharp. Hypomeron basally not produced ventrally and not sulcate	*Neoserica abnormis* group
8	Body surface strongly shiny. Body small: 5.7–6.6 mm	*Neoserica speciosa* group
8’	Body surface dull. Body larger 8 mm	*Chrysoserica* Brenske, 1897

### Key to species of the *Neoserica abnormis* group (males)

**Table:** 

1	Dorsal surface without numerous long semi-erect setae. Antennal club composed of 6 or 7 antennomeres	2
1’	Dorsal surface with numerous long semi-erect setae. Antennal club composed of 5 antennomeres	*Neoserica natmatoungensis* sp. n.
2	Odd intervals of elytra without white, scale-like setae. Antennal club composed of 6 antennomeres	3
2’	Odd intervals of elytra with sparse short, white, scale-like setae. Eyes small, ratio diameter/interocular width < 0.5. Antennal club composed of 6 or 7 antennomeres	7
3	Left paramere subdivided in three lobes	4
3’	Left paramere compact, without lobes	6
4	Lobes of left paramere directed all distally. Eyes large, ratio diameter/interocular width > 0.5	5
4’	Basal and median lobe of left paramere directed basally. Basal half of parameres subsymmetrical. Eyes small, ratio diameter/interocular width < 0.5	*Neoserica huangi* sp. n.
5	Body oblong-oval. Eyes smaller, ratio diameter/interocular width: 0.75. Dorsal lobe of left paramere at base bent laterally and evenly curved ventrally. Antennomere 5 subequal to half of length of club	*Neoserica trifida* sp. n.
5’	Body slender. Eyes larger, ratio diameter/interocular width: 0.92. Dorsal lobe of left paramere straight, parallel to the two remaining lobes of left paramere, abruptly curved ventrally at apex. Antennomere 5 subequal to length of club	*Neoserica yingjiangensis* sp. n.
6	Right paramere compact, without lobes	*Neoserica bairailingshanica* sp. n.
6’	Right paramere subdivided into a basal (dorsal) and distal (ventral) lobe	*Neoserica ponderosa* Arrow
7	Antennal club composed of 6 antennomeres	8
7’	Antennal club composed of 7 antennomeres	20
8	Left paramere long and slender	9
8’	Left paramere of various shape but not long and slender	10
9	Right paramere long and slender, phallobase without apical process	*Neoserica putaoana* sp. n.
9’	Right paramere extremely short. Phallobase with long lateral process on right apex	*Neoserica alloputaoana* sp. n.
10	Apical margin of elytra slightly concave before apical angle	*Neoserica thailandensis* sp. n.
10’	Apical margin of elytra slightly convex or straight before apical angle	11
11	Basal process of right paramere present	12
11’	Basal process of right paramere absent	*Neoserica simplicissima* sp. n.
12	Basal process of right paramere directed basally	14
12’	Basal process of right paramere directed distally	13
13	Left paramere in median cross section wider than high (e.g. flattened in lateral view). Antennal club twice as long as remaining antennomeres combined	*Neoserica kanphantensis* sp. n.
13’	Left paramere in median cross section higher than wide (e.g. flattened in dorsal view). Antennal club shorter	18
14	Left paramere trifid	15
14’	Left paramere bifid	16
15	Three lobes of left paramere of nearly same size	*Neoserica jiangxiensis* sp. n.
15’	Dorsal lobe of left paramere much smaller than other two	*Neoserica euyunnanica* sp. n.
16	Basal (i.e. dorsal) lobe of right paramere nearly half as long as distal one	*Neoserica tonkinea* sp. n.
16’	Basal (i.e. dorsal) lobe of right paramere much shorter than distal one	17
17	Ventral portion of right paramere (divided by strong bent) shorter than basal and dorsal part combined	*Neoserica taunggyiana* sp. n.
17’	Ventral portion of right paramere (divided by strong bent) subequal to basal and dorsal part combined	*Neoserica allolaotica* sp. n.
18	Dorsal (i.e. basal) lobe of right paramere small, less than a quarter of length of ventral (i.e. distal) one	*Neoserica abnormoides* sp. n.
18’	Dorsal (i.e. basal) lobe of right paramere large, subequal in size to ventral (i.e. distal) one	19
19	Dorsal lobe of left paramere large, subtriangular, distinctly wider than ventral one	*Neoserica abnormis* Moser
19’	Dorsal lobe of left paramere narrow, as wide as ventral one	*Neoserica yaoi* sp. n.
20	Parameres compact, not subdivided in lobes	*Neoserica lamellosa* sp. n.
20’	Parameres subdivided in lobes	21
21	Parameres strongly asymmetrical	*Neoserica cardamomensis* sp. n.
21’	Parameres nearly symmetrical	*Neoserica namthaensis* sp. n.

### Neoserica (s. l.) abnormis

Taxon classificationAnimaliaColeopteraScarabaeidae

Moser, 1908

Figs 1A–D , 8

Neoserica abnormis Moser, 1908: 329 [type locality: Vietnam, Mt. Mauson (Tonkin)].

#### Type material examined.

Lectotype (here designated): ♂ “Tonkin Montes Mauson April, Mai 2-3000’ H. Fruhstorfer / abnormis Mos. [handwritten Moser]” (ZMHB). Paralectotypes: 3 ♂♂, 3 ♀♀ “Tonkin Montes Mauson April, Mai 2-3000’ H. Fruhstorfer” (ZMHB).

#### Additional material examined.

1 ♂ “Tonkin Montes Mauson April, Mai 2-3000’ H. Fruhstorfer / Museum Paris ex. Coll. R. Oberthür / 95 Sericini: Asia spec.” (MNHN), 1 ♂ “N. Vietnam (Tonkin) Tamdao 12.–24.5.1989 Pacholátko leg.” (CPPB).

#### Redescription.

**Lectotype.** Body length: 13 mm, length of elytra: 9.6 mm, width: 7.2 mm. Body oblong, dark brown, antennal club brown, anterior labroclypeus shiny, dorsal surface dull, sparsely setose.

Labroclypeus subtrapezoidal, distinctly wider than long, widest at base, lateral margins moderately convex and convergent anteriorly, anterior angles moderately rounded, anterior margin weakly sinuate medially, margins moderately reflexed; surface nearly flat and shiny, basis with dull toment, punctation dense, anteriorly more sparse, behind the anterior margin with coarse punctures each bearing a long erect seta; frontoclypeal suture indistinctly incised, flat and distinctly curved medially; smooth area anterior to eye approximately 1.5 times as wide as long; ocular canthus moderately long (length = 1/3 of ocular diameter) and slender, glabrous, with a fine terminal seta. Frons dull, with fine and sparse punctures, beside the eyes with a few erect setae. Eyes small, ratio diameter/interocular width: 0.49. Antenna with ten antennomeres, club with six antennomeres, straight, only slightly longer than the remaining antennomeres combined; antennomere 4 slightly transverse, antennomere 3 half as long as pedicellus. Mentum elevated and slightly flattened anteriorly. Labrum distinctly produced medially, with a moderate median sinuation.

Pronotum moderately transverse, subtrapezoidal, widest at base, lateral margins evenly convex and strongly convergent anteriorly, anterior angles sharp and distinctly produced, posterior angles blunt, slightly rounded at the tip; anterior margin nearly straight, with a distinct and complete marginal line; surface densely and finely punctate with minute setae in punctures; setae of anterior and lateral border sparse; hypomeron basally distinctly carinate, but carina only weakly produced. Scutellum moderately long, triangular with convex sides and with the apex slightly rounded, with fine, moderately dense punctures, with only minute setae.

Elytra oblong, apex slightly truncate, widest shortly behind the middle, striae weakly impressed, finely and moderately densely punctate, intervals nearly flat, with moderately dense evenly spaced, fine punctures, intervals with a few fine white setae, otherwise only with very minute setae in punctures; epipleural edge fine, ending at the blunt external apical angle of elytra, epipleura sparsely setose, apical border chitinous, with only a very fine fringe of microtrichomes (visible at 100× magnification).

Ventral surface dull, coarsely and densely punctate, metasternum sparsely covered with setae on the disc, glabrous on sides; metacoxa glabrous, with a few short setae laterally, posterior margin weakly convex; abdominal sternites finely and unevenly densely punctuate, nearly glabrous, with a transverse row of coarse punctures, each bearing a robust short seta. Mesosternum between mesocoxae half as wide as slender mesofemur. Ratio of length of metepisternum/metacoxa: 1/1.79. Pygidium weakly convex and dull, coarsely and densely punctate, without smooth midline, with a few semi-erect setae beside the apical margin.

Legs slender; femora with two longitudinal rows of setae, finely and sparsely punctate between the rows; metafemur dull, anterior margin acute, behind anterior edge without serrated line, setae of anterior longitudinal row nearly completely lacking, posterior margin in apical half ventrally smooth and slightly widened, posterior margin dorsally distinctly serrated, on its basal portion with a few short setae. Metatibia slender and long, widest at apex, ratio of width/length: 1/3.5, sharply carinate dorsally, with two groups of spines, basal group just before the middle, apical group at three quarters of metatibial length, basally with a few robust but single setae; lateral face longitudinally convex, very finely, superficially and sparsely punctate, subdorsal longitudinal carina on lateral face present on about two third of metatibial length; ventral edge finely serrated, with three robust equidistant setae; medial face smooth, apex moderately concavely sinuate interiorly near tarsal articulation. Tarsomeres ventrally with sparse, short setae, laterally not carinate, protarsomeres smooth, meso- and metatarsomeres with a few very fine punctures; metatarsomeres ventrally glabrous, with a strongly serrated ridge ventrally and a sharp subventral carina immediately beside it, first metatarsomere slightly longer than following two tarsomeres combined and slightly longer than dorsal tibial spur. Protibia long, bidentate; anterior claws symmetrical, basal tooth of inner claw sharply truncate at apex.

**Figure 1.**
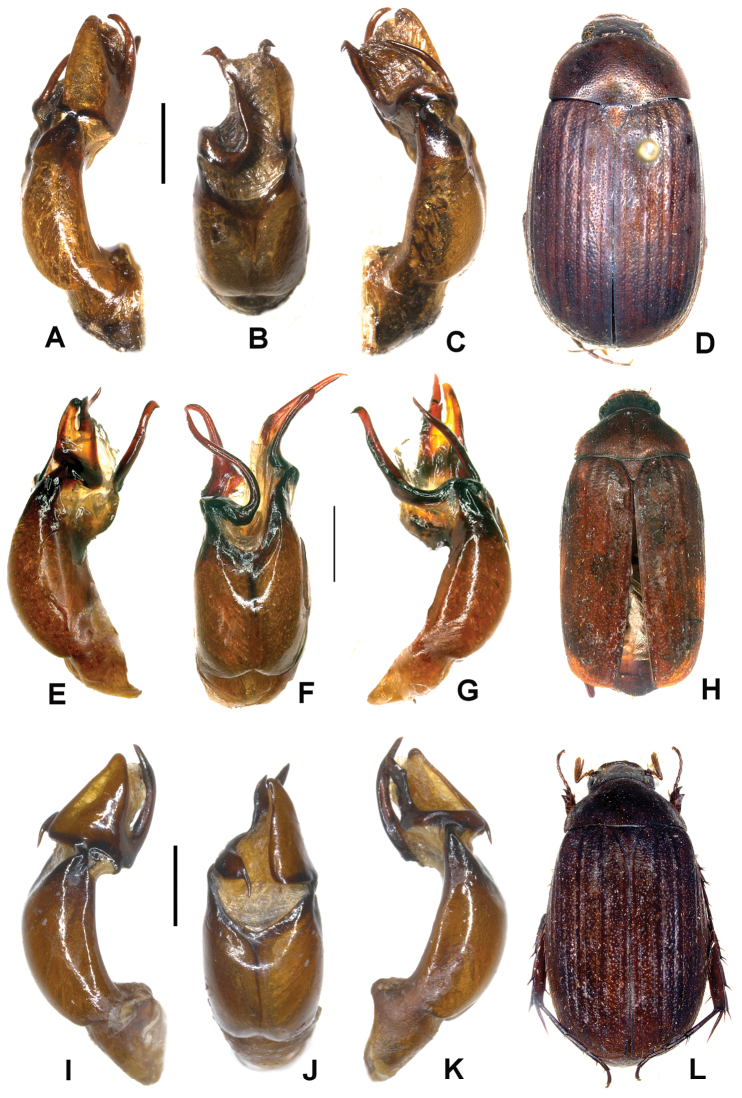
**A–D** *Neoserica abnormis* Moser (lectotype) **E–H** *Neoserica yaoi* sp. n. (holotype) **I–L** *Neoserica allolaotica* sp. n. (holotype) **A, E, I** Aedeagus, left side lateral view **C, G, K** Aedeagus, right side lateral view **B, F, J** parameres, dorsal view **D, H, L** Habitus. Scale: 1 mm. Habitus not to scale.

Aedeagus: Fig. 1A–C.

#### Variation.

Body length: 13–17 mm, length of elytra: 9.6–12 mm, width: 7.2–8.5 mm. Female: antennal club composed of four lamellae, as long as remaining antennomeres combined.

### Neoserica (s. l.) yaoi sp. n.

Taxon classificationAnimaliaColeopteraScarabaeidae

http://zoobank.org/1307EF7F-090A-45FE-A7CB-63AF950B20D5

Figs 1E–H , 8

#### Type material examined.

Holotype: ♂ “Defu, Napo, Guangxi, 19.VI.2000, 1350m, leg. Yao Jian” (IZAS). Paratypes: 1 ♂ “Defu, Napo, Guangxi, 19.VI.2000, 1350m, leg. Zhu Chaodong “ (ZFMK), 1 ♂ “Mengla, Yunnan, 21.IV.1982, leg. Jiang Shengqiao” (IZAS).

#### Description.

Body length: 14.4 mm, length of elytra: 10.3 mm, width: 6.9 mm. Body oblong, dark brown, antennal club brown, anterior labroclypeus shiny, dorsal surface dull, sparsely setose.

Labroclypeus subtrapezoidal, distinctly wider than long, widest at base, lateral margins moderately convex and convergent anteriorly, anterior angles moderately rounded, anterior margin weakly sinuate medially, margins moderately reflexed; surface nearly flat and shiny, basis with dull toment, punctation dense, anteriorly more sparse, behind the anterior margin with coarse punctures each bearing a long erect seta; frontoclypeal suture indistinctly incised, flat and distinctly curved medially; smooth area anterior to eye approximately 1.5 times as wide as long; ocular canthus moderately long (length = 1/3 of ocular diameter) and slender, glabrous, with a fine terminal seta. Frons dull, with fine and sparse punctures, beside the eyes with a few erect setae. Eyes small, ratio diameter/interocular width: 0.53. Antenna with ten antennomeres, club with six antennomeres, straight, 1.2 times as long as the remaining antennomeres combined; antennomere 4 slightly transverse, antennomere 3 half as long as pedicellus. Mentum elevated and slightly flattened anteriorly. Labrum distinctly produced medially, with a moderate median sinuation.

Pronotum moderately transverse, subtrapezoidal, widest at base, lateral margins weakly convex, convexly bent at middle, in basal half subparallel, in apical half strongly convergent anteriorly, anterior angles sharp and distinctly produced, posterior angles blunt, slightly rounded at the tip; anterior margin nearly straight, with a distinct and complete marginal line; surface densely and finely punctate with minute setae in punctures; setae of anterior and lateral border sparse; hypomeron basally distinctly carinate, but carina only weakly produced. Scutellum moderately long, triangular with convex sides and with the apex slightly rounded, with fine, moderately dense punctures, with only minute setae.

Elytra oblong, apex slightly truncate, widest at middle, striae weakly impressed, finely and moderately densely punctate, intervals nearly flat, with moderately dense evenly spaced, fine punctures, intervals with a few fine white setae, otherwise only with very minute setae in punctures; epipleural edge fine, ending at the blunt external apical angle of elytra, epipleura sparsely setose, apical border chitinous, with only a very fine fringe of microtrichomes (visible at 100× magnification).

Ventral surface dull, coarsely and densely punctate, metasternum sparsely covered with setae on the disc, glabrous on sides; metacoxa glabrous, with a few short setae laterally, posterior margin weakly convex; abdominal sternites finely and unevenly densely punctuate, nearly glabrous, with a transverse row of coarse punctures, each bearing a robust short seta. Mesosternum between mesocoxae half as wide as slender mesofemur. Ratio of length of metepisternum/metacoxa: 1/1.79. Pygidium weakly convex and dull, coarsely and densely punctate, without smooth midline, with a few semi-erect setae on apical half.

Legs slender; femora with two longitudinal rows of setae, finely and sparsely punctate between the rows; metafemur dull, anterior margin acute, behind anterior edge without serrated line, setae of anterior longitudinal row nearly completely lacking, posterior margin in apical half ventrally smooth and slightly widened, posterior margin dorsally distinctly serrated, on its basal portion with a few short setae. Metatibia slender and long, widest at apex, ratio of width/length: 1/3.7, sharply carinate dorsally, with two groups of spines, basal group just before the middle, apical group at three quarters of metatibial length, basally with a few robust but single setae; lateral face longitudinally convex, very finely, superficially and sparsely punctate, subdorsal longitudinal carina on lateral face present on about two third of metatibial length; ventral edge finely serrated, with three robust equidistant setae; medial face smooth, apex moderately concavely sinuate interiorly near tarsal articulation. Tarsomeres ventrally with sparse, short setae, laterally not carinate, protarsomeres smooth, meso- and metatarsomeres with a few very fine punctures dorsally; metatarsomeres ventrally glabrous, with a strongly serrated ridge ventrally and a sharp subventral carina immediately beside it, first metatarsomere slightly longer than following two tarsomeres combined and slightly longer than dorsal tibial spur. Protibia long, bidentate; anterior claws symmetrical, basal tooth of inner claw sharply truncate at apex.

Aedeagus: Fig. 1E–G. Female unknown.

#### Diagnosis.

The new species differs from the most closely related *Neoserica abnormis* by the narrow dorsal lobe of left paramere, which is as wide as the ventral lobe; in *Neoserica abnormis*, the dorsal lobe of left paramere is much wider than the ventral one.

#### Variation.

Body length: 13.5–14.4 mm, length of elytra: 10.0–10.3 mm, width: 6.5–6.9 mm.

#### Etymology.

The species is named after one of its collectors, Yao Jian.

### Neoserica (s. l.) allolaotica sp. n.

Taxon classificationAnimaliaColeopteraScarabaeidae

http://zoobank.org/19ACCAF9-59F9-40EE-9E83-229685F582FA

Figs 1I–L , 8

#### Type material examined.

Holotype: ♂ “N. Thailand, Doi Pui, near Chiang Mai; 09.V.1085; leg. H. Nara” (ZFMK). Paratypes: 1 ♂ “[BMNH] 703021 Laos, Phongsaly, Phongsaly env., 21°41.2'N, 102°6-8'E, leg. P. Pacholátko/ DA21/ =246 Sericini Asia” (ZFMK), 1 ♂ “Thai Chiang Mai prov. 18°49’N, 98°54'E 1600m, Doi Pui mt., 2.-6.v. Vit Kubáň leg., 1996/ Coll. P. Pacholátko Invt. No./ TS114” (CPPB), 8 ♂♂, 1 ♀ “N. Thailand, Doi Pui, near Chiang Mai; 09.V.1085; leg. H. Nara” (ZFMK), 39 ♂♂ “Laos-NE, Houa Phan prov., 20°13'09–19"N, 103°59'54''-104°00'03"E, 1480–1510m Phou Pane Mt., 22.IV.-14.V.2008 Vit Kubáň leg./ 875 Sericini Asia spec.” (ZFMK, NMPC), 4 ♂♂ “Laos-NE, Houa Phan prov., 20°13'09–19"N, 103°59'54"–104°00'03"E, 1480–1510m Phou Pane Mt., 22.4.–14.5.2008 Vit Kubáň leg./ 875 Sericini Asia spec.” (ZFMK, NMPC), 1 ♂ „NE-Laos: Hua Phan prov., Ban Saleui, Phou Pan (Mt.) - 20°12'N, 104°01'E; 14.iv.–15.v.2012; 1300–1900m; leg. C. Holzschuh Ankauf ZFMK Bonn 2012/13“ (ZFMK), 2 ♂♂ “Laos-NE Hua Phan prov., 20°12'N, 104°01'E, Phu Phan Mt., 1500–1900m, 17.5.–3.6.2007, leg. C. Holzschuh” (ZFMK), 4 ♂♂ “Laos-NE Hua Phan prov., 20°12'N, 104°01'E, Phu Phan Mt., 1500–1900m, 17.5.–3.6.2007, leg. Vit Kuban” (ZFMK), 8 ♂♂ “Laos-NE, Houa Phan prov., 20°13'09–19"N, 103°59'54"-104°00'03"E, 1480–1550m Phou Pane Mt., 9.–16.vi.2009, David Hauck leg./ NHMB Basel, NMPC Prague Laos 2009 Expedition: M. Brancucci, M. Geiser, Z. Kraus, D. Hauck, V. Kubáň” (NHMB), 16 ♂♂ “Laos-NE, Houa Phan prov., 20°13'09–19"N, 103°59'54"-104°00'03"E, 1480–1550m Phou Pane Mt., 1.–16.vi.2009, Zdenek Kraus leg./ NHMB Basel, NMPC Prague Laos 2009 Expedition: M. Brancucci, M. Geiser, Z. Kraus, D. Hauck, V. Kubáň” (NHMB), 11 ♂♂ “Laos-NE, Houa Phan prov., ~20°13'N, 104°00'E, Phou Pane Mt., 1.–16.vi.2009, 1350–1500 m, M. Brancucci leg./ NHMB Basel, NMPC Prague Laos 2009 Expedition: M. Brancucci, M. Geiser, Z. Kraus, D. Hauck, V. Kubáň” (NHMB), 1 ♂ “Laos-NE, Houa Phan prov., ~20°12–13.5'N, 103°59.5'–104°01'E, Ban Saluei - Phou Pane Mt., 10.-16.vi.2009, 1340–1870 m, M. Brancucci & local collectors leg./ NHMB Basel, NMPC Prague Laos 2009 Expedition: M. Brancucci, M. Geiser, Z. Kraus, D. Hauck, V. Kubáň” (NHMB), 3 ♂♂ “Laos-NE, Houa Phan prov., ~20°12–13.5'N, 103°59.5'–104°01'E, Ban Sauei - Phou Pane Mt., 1340–1870 m, 15.iv–15.v.2008, Lao collectors leg.” (NHMB), 30 ♂♂ “Laos-NE, Houa Phan prov., 20°11–13'N, 103°59’-104°01'E, Ban Sauei - Phou Pane Mt., 9.–17.vi.2009, 1300–1900 m, Michael Geiser leg. / NHMB Basel, NMPC Prague Laos 2009 Expedition: M. Brancucci, M. Geiser, Z. Kraus, D. Hauck, V. Kubáň” (NHMB), 1 ♂ “Laos-NE, Xieng Khouang prov., 19°38.20'N, 103°20.20'E, Phonsavan (30 km NE); Phou Sane Mt., 1420 m, 10.–30.v.2009, D. Hauck leg. / NHMB Basel, NMPC Prague Laos 2009 Expedition: M. Brancucci, M. Geiser, Z. Kraus, D. Hauck, V. Kubáň” (NHMB), 11 ♂♂ “Laos-NE, Xieng Khouang prov., 19°38.20'N, 103°20.20'E, Phonsavan (30 km NE): Phou Sane Mt., 1420 m, 10.–30.v.2009, Z. Kraus leg. / NHMB Basel, NMPC Prague Laos 2009 Expedition: M. Brancucci, M. Geiser, Z. Kraus, D. Hauck, V. Kubáň” (NHMB), 9 ♂♂ “Laos-NE, Xieng Khouang prov., 19°37–38'N, 103°20'E, 30 km NE Phonsavan: Ban Na Lam - Phou Sane Mt., 1300–1500 m, 10.-30.v.2009, M. Brancucci leg. / NHMB Basel, NMPC Prague Laos 2009 Expedition: M. Brancucci, M. Geiser, Z. Kraus, D. Hauck, V. Kubáň” (NHMB), 15 ♂♂ “Laos-NE, Xieng Khouang prov., 19°37–38'N, 103°20'E, 30 km NE Phonsavan: Ban Na Lam - Phou Sane Mt., 1300–1500 m, 10.–30.v.2009, M. Geiser leg. / NHMB Basel, NMPC Prague Laos 2009 Expedition: M. Brancucci, M. Geiser, Z. Kraus, D. Hauck, V. Kubáň” (NHMB).

#### Description.

Body length: 12.3 mm, length of elytra: 9.6 mm, width: 7.4 mm. Body oblong, dark brown, antennal club yellowish brown, anterior labroclypeus shiny, dorsal surface dull, opaque toment on elytra and pronotum less thick, with a light trace of shine, sparsely setose.

Labroclypeus slightly subtrapezoidal, distinctly wider than long, widest at base, lateral margins moderately convex and convergent anteriorly, anterior angles strongly rounded, anterior margin distinctly sinuate medially, margins moderately reflexed; surface slightly convex and shiny, basis with dull toment, punctation dense, anteriorly more sparse, behind the anterior margin with coarse punctures each bearing a long erect seta; frontoclypeal suture distinctly incised, flat and distinctly curved medially; smooth area anterior to eye approximately 1.5 times as wide as long; ocular canthus moderately long (length = 1/3 of ocular diameter) and slender, glabrous, with a fine terminal seta. Frons dull, with fine and sparse punctures, beside the eyes with a few erect setae. Eyes small, ratio diameter/interocular width: 0.49. Antenna with ten antennomeres, club with six antennomeres, straight, 1.5 times as long as remaining antennomeres combined; antennomere 5 subequal to length of club, antennomere 4 distinctly transverse, antennomere 3 half as long as pedicellus. Mentum elevated and slightly flattened anteriorly. Labrum distinctly produced medially, with a moderate median sinuation.

Pronotum moderately transverse, subtrapezoidal, widest at base, lateral margins evenly convex and strongly convergent anteriorly, anterior angles sharp and distinctly produced, posterior angles blunt, distinctly rounded at the tip; anterior margin nearly straight, with a fine and complete marginal line; surface densely and finely punctate with minute setae in punctures; setae of anterior and lateral border sparse; hypomeron basally distinctly carinate, but carina only weakly produced. Scutellum moderately long, triangular with nearly straight sides, apex slightly rounded, with fine, dense punctures, with only minute setae.

Elytra oblong, apex slightly truncate, widest shortly behind the middle, striae weakly impressed, finely and moderately densely punctate, odd intervals narrower and distinctly convex with punctures concentrated along the striae, others evenly punctate and nearly flat, odd intervals with white scale-like, adpressed setae, otherwise only with very minute setae in punctures; epipleural edge fine, very narrow behind the middle, ending at the blunt external apical angle of elytra, epipleura only sparsely setose, apical border chitinous, with only a very fine fringe of microtrichomes (visible at 100× magnification).

Ventral surface dull, coarsely and densely punctate, metasternum sparsely covered with setae on the disc, glabrous on sides; metacoxa glabrous, with a few short setae laterally, posterior margin weakly convex; abdominal sternites finely and unevenly densely punctuate, nearly glabrous, with a transverse row of coarse punctures, each bearing a robust short seta. Mesosternum between mesocoxae half as wide as slender mesofemur. Ratio of length of metepisternum/metacoxa: 1/1.67. Pygidium weakly convex and dull, densely punctate, fine punctures mixed with coarser ones, without smooth midline, with a few semi-erect setae basally on sides, at apex with short, fine, moderately dense setae.

Legs slender; femora with two longitudinal rows of setae, finely and sparsely punctate between the rows; metafemur dull, anterior margin acute, behind anterior edge without serrated line, setae of anterior longitudinal row nearly completely lacking, posterior margin in apical half ventrally smooth and slightly widened, posterior margin dorsally distinctly serrated, on its basal portion with a few short setae. Metatibia slender and long, widest at apex, ratio of width/length: 1/3.9, sharply carinate dorsally, with two groups of spines, basal group just before the middle, apical group at three quarters of metatibial length, basally with a few robust but single setae; lateral face longitudinally convex, very finely, superficially and sparsely punctate, subdorsal longitudinal carina on lateral face present on about two third of metatibial length; ventral edge finely serrated, with four robust equidistant setae; medial face smooth, apex moderately concavely sinuate interiorly near tarsal articulation. Tarsomeres ventrally with sparse, short setae, laterally not carinate, protarsomeres smooth, meso- and metatarsomeres with a few very fine punctures; metatarsomeres ventrally glabrous, with a strongly serrated ridge ventrally and a sharp subventral carina immediately beside it, first metatarsomere slightly longer than following two tarsomeres combined and slightly longer than dorsal tibial spur. Protibia long, bidentate; anterior claws symmetrical, basal tooth of inner claw sharply truncate at apex.

Aedeagus: Fig. 1I–K.

#### Diagnosis.

*Neoserica allolaotica* is similar to *Neoserica taunggyiana* externally and in the general shape of male genitalia. It differs in the shape of the parameres: the ventral lobe of left paramere is subtrapezoidal (in lateral view) and truncate at apex, and the ventral portion (distal to the strong bent) of right paramere is subequal in length to the basal and dorsal portion, while in *Neoserica taunggyiana* it is shorter.

For the paratype specimen with BMNH code 703021 DNA data are available for the markers Cox1, 16S, and 28S under Genbank accession numbers EU084100, EF487898, and EU084238, respectively.

#### Variation.

Body length: 12.3–14.4 mm, length of elytra: 9.6–10.4 mm, width: 7.4–7.6 mm.

#### Etymology.

The new species is named according to its occurrence in Laos (‘*laotica*’) with the [Greek] prefix *allo*- (different, other) to avoid potential secondary homonymy with *Neoserica laotica* Frey.

#### Remarks.

As the species occurs syntopically with several other species (in particular with *Neoserica cardamomensis*) in its distribution range, it was not possible to assign female specimens to the type series unambiguously based on morphological characters alone. They were therefore omitted from species description so far.

### Neoserica (s. l.) tonkinea sp. n.

Taxon classificationAnimaliaColeopteraScarabaeidae

http://zoobank.org/474E9936-D7F4-4B2E-A780-13379E92D641

Figs 2A–E , 8

#### Type material examined.

Holotype: ♂ “N Vietnam (Tonkin) pr. Vinh Phu 1990 Tam Dao 6.–9.v. P. Pacholátko leg./ Coll. P. Pacholátko Invt. No./ VS74” (CPPB). Paratypes: 3 ♂♂ “Vietnam N (Sa Pa) Lao Cai Prov., 250km from Hanoi bearing 31°, Sa Pa vill. Env. Hoang Lien Son Nat. Res. 27.5.–3.6.1998 1250m leg. A. Napolov” (CNAR, ZFMK), 1 ♂ “Vietnam N (Sa Pa) Lao Cai Prov., 250km from Hanoi bearing 31°, Sa Pa vill. Env. Hoang Lien Son Nat. Res. 21.–23.6.1998 1250m leg. A. Napolov” (CNAR).

#### Description.

Body length: 13.0 mm, length of elytra: 9.8 mm, width: 7.8 mm. Body oblong, dark brown, antennal club yellowish brown, anterior labroclypeus shiny, dorsal surface dull, opaque toment on elytra and pronotum less thick, with a light trace of shine, sparsely setose.

Labroclypeus slightly subtrapezoidal, distinctly wider than long, widest at base, lateral margins moderately convex and convergent anteriorly, anterior angles strongly rounded, anterior margin weakly sinuate medially, margins moderately reflexed; surface slightly convex and shiny, basis with dull toment, punctation dense, anteriorly more sparse, behind the anterior margin with coarse punctures each bearing a long erect seta; frontoclypeal suture distinctly incised, flat and distinctly curved medially; smooth area anterior to eye approximately 1.5 times as wide as long; ocular canthus moderately wide and moderately long (length = 1/3 of ocular diameter), glabrous, with a fine terminal seta. Frons dull, with fine and dense punctures, beside the eyes and the frontoclypeal suture with a few erect setae. Eyes small, ratio diameter/interocular width: 0.48. Antenna with ten antennomeres, club with six antennomeres, straight, 1.2 times as long as remaining antennomeres combined; antennomere 5 subequal to length of club, antennomere 4 slightly transverse, antennomere 3 half as long as pedicellus. Mentum elevated and slightly flattened anteriorly. Labrum distinctly produced medially, with a moderate median sinuation.

Pronotum moderately transverse, subtrapezoidal, widest at base, lateral margins evenly convex and convergent anteriorly, anterior angles sharp and distinctly produced, posterior angles blunt, strongly rounded at the tip; anterior margin nearly straight, with a fine and complete marginal line; surface densely and finely punctate with minute setae in punctures; setae of lateral border sparse; hypomeron basally distinctly carinate, but carina only weakly produced. Scutellum long, triangular with nearly straight sides, apex slightly rounded, with fine, dense punctures, basally impunctate at middle, with only minute setae.

Elytra oblong, widest shortly behind the middle, striae weakly impressed, finely and moderately densely punctate, odd intervals narrower and distinctly convex with punctures concentrated along the striae, others evenly punctate and nearly flat, odd intervals with white scale-like, adpressed setae, otherwise only with very minute setae in punctures; epipleural edge fine, very narrow behind the middle, ending at the moderately rounded external apical angle of elytra, epipleura densely setose, apical border chitinous, with only a very fine fringe of microtrichomes (visible at 100× magnification).

Ventral surface dull, coarsely and densely punctate, metasternum sparsely covered with setae on the disc, glabrous on sides; metacoxa glabrous, with a few short setae laterally, posterior margin weakly convex; abdominal sternites finely and unevenly densely punctuate, nearly glabrous, with a transverse row of coarse punctures, each bearing a robust short seta. Mesosternum between mesocoxae half as wide as slender mesofemur. Ratio of length of metepisternum/metacoxa: 1/1.77. Pygidium moderately convex and dull, densely punctate, fine punctures mixed with coarser ones, without smooth midline, with a few setae beside the margin.

Legs slender; femora with two longitudinal rows of setae, finely and sparsely punctate between the rows; metafemur dull, anterior margin acute, behind anterior edge without serrated line, setae of anterior longitudinal row nearly completely lacking, posterior margin in apical half ventrally smooth and slightly widened, posterior margin dorsally distinctly serrated, on its basal portion with a few short setae. Metatibia slender and long, widest at apex, ratio of width/length: 1/3.7, sharply carinate dorsally, with two groups of spines, basal group just before the middle, apical group at three quarters of metatibial length, basally with a few robust but single setae; lateral face longitudinally convex, very finely, superficially and sparsely punctate, subdorsal longitudinal carina on lateral face present on about two third of metatibial length; ventral edge finely serrated, with four robust equidistant setae; medial face smooth, apex moderately concavely sinuate interiorly near tarsal articulation. Tarsomeres ventrally with sparse, short setae, laterally not carinate, protarsomeres smooth, mesotarsomeres with a few very fine punctures; metatarsomeres lacking in holotype. Protibia long, bidentate; anterior claws symmetrical, basal tooth of inner claw sharply truncate at apex.

**Figure 2.**
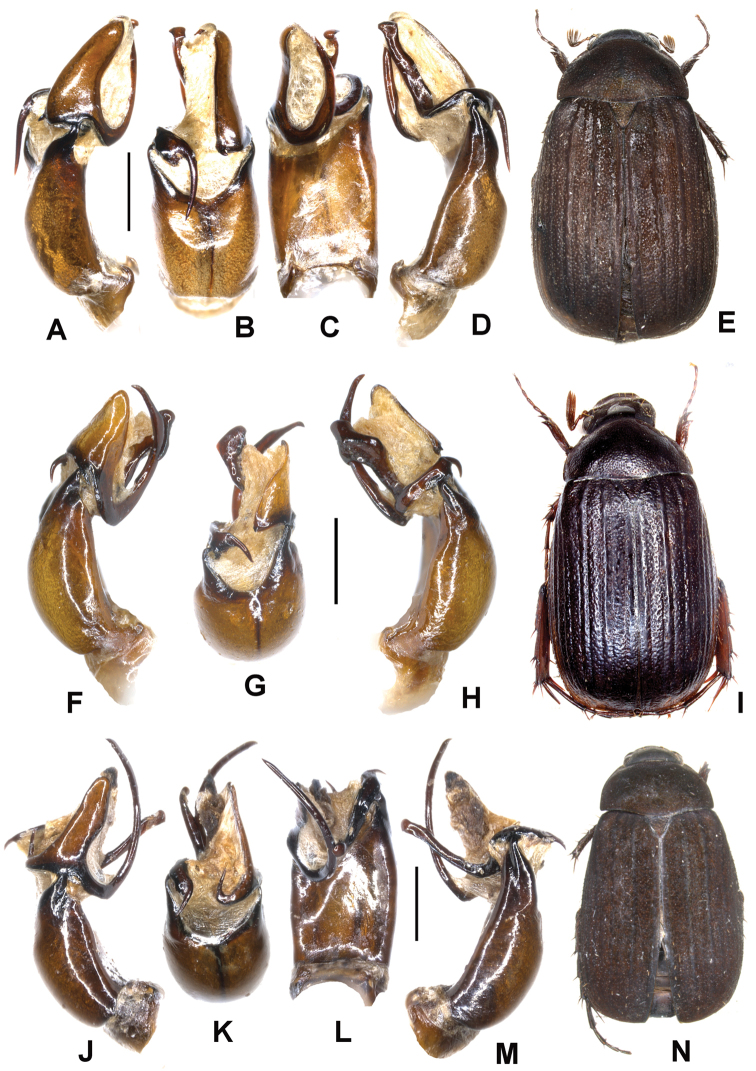
**A–E** *Neoserica tonkinea* sp. n. (holotype), **F–I** *Neoserica taunggyiana* sp. n. (holotype) **J–N** *Neoserica euyunnanica* sp. n. (holotype) **A, F, J** Aedeagus, left side lateral view **D, H, M** Aedeagus, right side lateral view **B, G, K** parameres, dorsal view **C, L** aedeagus, ventral view **E, I, N** Habitus. Scale: 1 mm. Habitus not to scale.

Aedeagus: Fig. 2A–D. Female unknown.

#### Diagnosis.

*Neoserica tonkinea* sp. n. is very similar to *Neoserica allolaotica* and *Neoserica taunggyiana* externally and in the general shape of male genitalia. It differs principally by the shape of the parameres: the dorsal lobe of left paramere is narrower than in *Neoserica allolaotica* and the ventral lobe is not extended basally as in *Neoserica taunggyiana*; the right paramere is much longer and less widened apically than in either of the species (in lateral view), the basal lobe is directed basally as well but much longer being more than half as long as the distal portion of the right paramere.

#### Variation.

Body length: 12.5–13.0 mm, length of elytra: 9.4–9.8 mm, width: 7.2–7.8 mm.

#### Etymology.

The new species is named according to its occurrence in northern Vietnam, formerly during colonial times, called Tonkin.

### Neoserica (s. l.) taunggyiana sp. n.

Taxon classificationAnimaliaColeopteraScarabaeidae

http://zoobank.org/7A8ABD11-3E21-4D8A-9544-B5FE998A67CC

Figs 2F–I , 9

#### Type material examined.

Holotype: ♂ “Burma (Myanmar) SW Shan state Taunggyi J. Rejsek 1.–18.6.1997/ Coll. Dirk Ahrens” (ZFMK).

#### Description.

Body length: 12.0 mm, length of elytra: 8.9 mm, width: 6.6 mm. Body oblong, dark brown, antennal club yellowish brown, anterior labroclypeus shiny, dorsal surface dull, opaque toment on elytra and pronotum less thick, with a light trace of shine, sparsely setose.

Labroclypeus slightly subtrapezoidal, distinctly wider than long, widest at base, lateral margins moderately convex and convergent anteriorly, anterior angles strongly rounded, anterior margin distinctly sinuate medially, margins moderately reflexed; surface slightly convex and shiny, basis with dull toment, punctation dense, anteriorly more sparse, behind the anterior margin with coarse punctures each bearing a long erect seta; frontoclypeal suture distinctly incised, flat and distinctly curved medially; smooth area anterior to eye approximately 1.5 times as wide as long; ocular canthus moderately long (length = 1/3 of ocular diameter) and slender, glabrous, with a fine terminal seta. Frons dull, with fine and sparse punctures, beside the eyes with a few erect setae. Eyes small, ratio diameter/interocular width: 0.52. Antenna with ten antennomeres, club with six antennomeres, straight, 1.2 times as long as remaining antennomeres combined; antennomere 5 subequal to length of club, antennomere 4 distinctly transverse, antennomere 3 half as long as pedicellus. Mentum elevated and slightly flattened anteriorly. Labrum distinctly produced medially, with a moderate median sinuation.

Pronotum moderately transverse, subtrapezoidal, widest at base, lateral margins evenly convex in the basal half, in anterior third nearly straight, throughout strongly convergent anteriorly, anterior angles very sharp and distinctly produced, posterior angles blunt, slightly rounded at the tip; anterior margin nearly straight, with a fine and complete marginal line; surface densely and finely punctate with minute setae in punctures; setae of anterior and lateral border sparse; hypomeron basally distinctly carinate, but carina only weakly produced. Scutellum moderately long, triangular with nearly straight sides, apex slightly rounded, with fine, dense punctures, with only minute setae.

Elytra oblong, apex slightly truncate, widest shortly behind the middle, striae weakly impressed, finely and moderately densely punctate, odd intervals narrower and distinctly convex with punctures concentrated along the striae, others evenly punctate and nearly flat, odd intervals with white scale-like, adpressed setae, otherwise only with very minute setae in punctures; epipleural edge fine, very narrow behind the middle, ending at the blunt external apical angle of elytra, epipleura only sparsely setose, apical border chitinous, with only a very fine fringe of microtrichomes (visible at 100× magnification).

Ventral surface dull, coarsely and densely punctate, metasternum sparsely covered with setae on the disc, glabrous on sides; metacoxa glabrous, with a few short setae laterally, posterior margin weakly convex; abdominal sternites finely and unevenly densely punctuate, nearly glabrous, with a transverse row of coarse punctures, each bearing a robust short seta. Mesosternum between mesocoxae half as wide as slender mesofemur. Ratio of length of metepisternum/metacoxa: 1/1.7. Pygidium weakly convex and dull, densely punctate, fine punctures mixed with coarser ones, without smooth midline, with a few semi-erect setae basally on sides, at apex with short, fine, moderately dense setae.

Legs slender; femora with two longitudinal rows of setae, finely and sparsely punctate between the rows; metafemur dull, anterior margin acute, behind anterior edge without serrated line, setae of anterior longitudinal row nearly completely lacking, posterior margin in apical half ventrally smooth and slightly widened, posterior margin dorsally distinctly serrated, on its basal portion with a few short setae. Metatibia slender and long, widest at apex, ratio of width/length: 1/3.9, sharply carinate dorsally, with two groups of spines, basal group just before the middle, apical group at three quarters of metatibial length, basally with a few robust but single setae; lateral face longitudinally convex, very finely, superficially and sparsely punctate, subdorsal longitudinal carina on lateral face present on about two third of metatibial length; ventral edge finely serrated, with four robust equidistant setae; medial face smooth, apex moderately concavely sinuate interiorly near tarsal articulation. Tarsomeres ventrally with sparse, short setae, laterally not carinate, protarsomeres smooth, meso- and metatarsomeres with a few very fine punctures; metatarsomeres ventrally glabrous, with a strongly serrated ridge ventrally and a sharp subventral carina immediately beside it, first metatarsomere slightly longer than following two tarsomeres combined and slightly longer than dorsal tibial spur. Protibia long, bidentate; anterior claws symmetrical, basal tooth of inner claw sharply truncate at apex.

Aedeagus: Fig. 2F–H. Female unknown.

#### Diagnosis.

*Neoserica taunggyiana* is similar to *Neoserica abnormis* externally and in the general shape of male genitalia. It differs by the straight anterior margins of pronotum and, principally, by the shape of the parameres: the ventral lobe of left paramere is much longer and the basal (dorsal) lobe of right paramere is directed basally, rather than distally.

#### Etymology.

The new species is named according to the type locality, Taunggyi.

### Neoserica (s. l.) euyunnanica sp. n.

Taxon classificationAnimaliaColeopteraScarabaeidae

http://zoobank.org/86938D3C-EF12-49CB-8206-E4E652F2B271

Figs 2J–N , 8

#### Type material examined.

Holotype: ♂ “China: E-Yunnan Damaidi 2500m, Guangnan near Vietnam VII-2003 leg. Li et al.” (ZFMK). Paratypes: 1 ♂ “China, SE Yunnan, Xichou – E env., 1400-1700m, 13.–18.5.95 23°22-26'[N]/ 104°41-49'[E] L.+R. Businský lgt.” (CPPB), 1 ♂, 1 ♀ “CH, Guizhou prov., ~650m, Jiangkou (ca 50km SW), 27°32.83'N, 108°36.45'E, Shidu vill. env., 29.vi.-6.vii.2001, C. Holzschuh leg.” (CPPB).

#### Description.

Body length: 13.0 mm, length of elytra: 10.0 mm, width: 8.0 mm. Body oblong, dark brown, antennal club yellowish brown, anterior labroclypeus shiny, dorsal surface dull, opaque toment on elytra and pronotum less thick, with a light trace of shine, sparsely setose.

Labroclypeus slightly subtrapezoidal, distinctly wider than long, widest at base, lateral margins moderately convex and convergent anteriorly, anterior angles strongly rounded, anterior margin weakly sinuate medially, margins moderately reflexed; surface slightly convex and shiny, basis with dull toment, punctation dense, anteriorly more sparse, behind the anterior margin with coarse punctures each bearing a long erect seta; frontoclypeal suture distinctly incised, flat and distinctly curved medially; smooth area anterior to eye approximately 1.5 times as wide as long; ocular canthus moderately wide and moderately long (length = 1/3 of ocular diameter), glabrous, with a robust terminal seta. Frons dull, with fine and dense punctures, beside the eyes and behind the frontoclypeal suture with a few erect setae. Eyes small, ratio diameter/interocular width: 0.48. Antenna with ten antennomeres, club with six antennomeres, straight, 1.1 times as long as remaining antennomeres combined; antennomere 5 subequal to length of club, antennomere 4 slightly transverse, antennomere 3 half as long as pedicellus. Mentum elevated and slightly flattened anteriorly. Labrum distinctly produced medially, with a moderate median sinuation.

Pronotum moderately transverse, subtrapezoidal, widest at base, lateral margins evenly convex and convergent anteriorly, anterior angles sharp and distinctly produced, posterior angles blunt, slightly rounded at the tip; anterior margin nearly straight, with a fine and complete marginal line; surface densely and finely punctate with minute setae in punctures; setae of lateral border abrased in holotype; hypomeron basally distinctly carinate, but carina only weakly produced. Scutellum long, triangular with nearly straight sides, apex slightly rounded, with fine, dense punctures, basally impunctate at middle, with only minute setae.

Elytra oblong, widest shortly behind the middle, striae weakly impressed, finely and moderately densely punctate, odd intervals narrower and distinctly convex with punctures concentrated along the striae, others evenly punctate and nearly flat, odd intervals with white scale-like, adpressed setae, otherwise only with very minute setae in punctures; epipleural edge fine, very narrow behind the middle, ending at the moderately rounded external apical angle of elytra, epipleura only sparsely setose, apical border chitinous, with only a very fine fringe of microtrichomes (visible at 100× magnification).

Ventral surface dull, coarsely and densely punctate, metasternum sparsely covered with setae on the disc, glabrous on sides; metacoxa glabrous, with a few short setae laterally, posterior margin weakly convex; abdominal sternites finely and unevenly densely punctuate, nearly glabrous, with a transverse row of coarse punctures, each bearing a robust short seta. Mesosternum between mesocoxae half as wide as slender mesofemur. Ratio of length of metepisternum/metacoxa: 1/1.73. Pygidium moderately convex and dull, densely punctate, fine punctures mixed with coarser ones, without smooth midline, with numerous long setae on apex, otherwise with minute setae in punctures.

Legs slender; femora with two longitudinal rows of setae, finely and sparsely punctate between the rows; metafemur dull, anterior margin acute, behind anterior edge without serrated line, setae of anterior longitudinal row nearly completely lacking, posterior margin in apical half ventrally smooth and slightly widened, posterior margin dorsally distinctly serrated, on its basal portion with a few short setae. Metatibia slender and long, widest at apex, ratio of width/length: 1/3.8, sharply carinate dorsally, with two groups of spines, basal group just before the middle, apical group at three quarters of metatibial length, basally with a few robust but single setae; lateral face longitudinally convex, very finely, superficially and sparsely punctate, subdorsal longitudinal carina on lateral face present on about two third of metatibial length; ventral edge finely serrated, with four robust equidistant setae; medial face smooth, apex moderately concavely sinuate interiorly near tarsal articulation. Tarsomeres ventrally with sparse, short setae, laterally not carinate, protarsomeres smooth, meso- and metatarsomeres with a few very fine punctures; metatarsomeres ventrally glabrous, with a strongly serrated ridge ventrally and a sharp subventral carina immediately beside it, first metatarsomere slightly longer than following two tarsomeres combined and slightly longer than dorsal tibial spur. Protibia long, bidentate; anterior claws symmetrical, basal tooth of inner claw sharply truncate at apex.

Aedeagus: Fig. 2J–N.

#### Diagnosis.

*Neoserica euyunnanica* sp. n. is very similar to the previous three species externally and in the general shape of male genitalia. It differs by the shape of the parameres: the ventral lobe of left paramere is extended distally far beyond the apex of the dorsal lobe which produces dorsally a long hook, that is rudimentally present also in *Neoserica tonkinea* as a small sharply pointed tooth; the dorsal lobe of right paramere is slightly shorter than in *Neoserica tonkinea*.

#### Variation.

Body length: 13.0–13.5 mm, length of elytra: 10.0–10.3 mm, width: 8.0–9.0 mm. Female: Antennal club composed of four antennomeres, as long as remaining antennomeres combined.

#### Etymology.

The new species is named “*euyunnanica*” according to its occurrence in Yunnan (China).

### Neoserica (s. l.) jiangxiensis sp. n.

Taxon classificationAnimaliaColeopteraScarabaeidae

http://zoobank.org/55031ED2-31BA-4024-8452-62641AF4716B

Figs 3A–D , 8

#### Type material examined.

Holotype: ♂ “China, W-Jiangxi Jingang Shan- Ciping 2–14.VI.1994 E. Jendek & O. Šauša leg./ Coll. P. Pacholátko Invt. No./ CS10” (CPPB). Paratypes: 2 ♂♂ “China, W-Jiangxi Jingang Shan- Ciping 2-14.VI.1994 E. Jendek & O. Šauša leg.” (CP, ZFMK), 1 ♂ “Tongzhong Forestry Farm, Fangcheng, Guangxi, 9.IV.2002, light trap, leg. Xue Huaijun” (NKUT).

#### Description.

Body length: 14.0 mm, length of elytra: 10.5 mm, width: 8.2 mm. Body oblong, dark brown, antennal club yellowish brown, anterior labroclypeus shiny, dorsal surface dull, opaque toment on elytra and pronotum less thick, with a light trace of shine, sparsely setose.

Labroclypeus slightly subtrapezoidal, distinctly wider than long, widest at base, lateral margins nearly straight and convergent anteriorly, anterior angles strongly rounded, anterior margin distinctly sinuate medially, margins moderately reflexed; surface slightly convex and shiny, basis with dull toment, punctation dense, anteriorly more sparse, behind the anterior margin with coarse punctures each bearing a long erect seta; frontoclypeal suture distinctly incised, flat and distinctly curved medially; smooth area anterior to eye approximately 1.5 times as wide as long; ocular canthus moderately wide and moderately long (length = 1/3 of ocular diameter), glabrous, with one or two robust terminal setae. Frons dull, with fine and dense punctures, beside the eyes and behind the frontoclypeal suture with a few erect setae. Eyes small, ratio diameter/interocular width: 0.47. Antenna with ten antennomeres, club with six antennomeres, straight, as long as remaining antennomeres combined; antennomere 5 distinctly shorter than the club, antennomere 4 slightly widened but not transverse, antennomere 3 half as long as pedicellus. Mentum elevated and slightly flattened anteriorly. Labrum distinctly produced medially, with a moderate median sinuation.

Pronotum moderately transverse, subtrapezoidal, widest just before base, lateral margins evenly convex and convergent anteriorly, slightly convergent also towards the strongly rounded posterior angles, anterior angles sharp and distinctly produced; anterior margin nearly straight, with a fine and complete marginal line; surface densely and finely punctate with minute setae in punctures; setae of lateral border sparse; hypomeron basally distinctly carinate, but carina only weakly produced. Scutellum moderately long, triangular with nearly straight sides, apex slightly rounded, with fine, dense punctures, with only minute setae.

Elytra oblong, widest shortly behind the middle, striae weakly impressed, finely and moderately densely punctate, odd intervals narrower and slightly convex with punctures concentrated along the striae, others evenly punctate and nearly flat, odd intervals with white scale-like, adpressed setae, otherwise only with very minute setae in punctures; epipleural edge fine, very narrow behind the middle, ending at the moderately rounded external apical angle of elytra, epipleura only sparsely setose, apical border chitinous, with only a very fine fringe of microtrichomes (visible at 100× magnification).

Ventral surface dull, coarsely and densely punctate, metasternum sparsely covered with setae on the disc, glabrous on sides; metacoxa glabrous, with a few short setae laterally, posterior margin weakly convex; abdominal sternites finely and unevenly densely punctuate, nearly glabrous, with a transverse row of coarse punctures, each bearing a robust short seta. Mesosternum between mesocoxae half as wide as slender mesofemur. Ratio of length of metepisternum/metacoxa: 1/1.84. Pygidium apically strongly convex and dull, densely punctate, fine punctures mixed with coarser ones, without smooth midline, with numerous long setae on apex, otherwise with minute setae in punctures.

Legs slender; femora with two longitudinal rows of setae, finely and sparsely punctate between the rows; metafemur dull, anterior margin acute, behind anterior edge without serrated line, setae of anterior longitudinal row nearly completely lacking, posterior margin in apical half ventrally smooth and slightly widened, posterior margin dorsally distinctly serrated, on its basal portion with a few short setae. Metatibia slender and long, widest at apex, ratio of width/length: 1/3.9, sharply carinate dorsally, with two groups of spines, basal group just before the middle, apical group at three quarters of metatibial length, basally with a few robust but single setae; lateral face longitudinally convex, very finely, superficially and sparsely punctate, subdorsal longitudinal carina on lateral face present on about two third of metatibial length; ventral edge finely serrated, with four robust equidistant setae; medial face smooth, apex moderately concavely sinuate interiorly near tarsal articulation. Tarsomeres ventrally with sparse, short setae, laterally not carinate, protarsomeres smooth, meso- and metatarsomeres with a few very fine punctures; metatarsomeres ventrally glabrous, with a strongly serrated ridge ventrally and a sharp subventral carina immediately beside it, first metatarsomere slightly longer than following two tarsomeres combined and slightly longer than dorsal tibial spur. Protibia long, bidentate; anterior claws symmetrical, basal tooth of inner claw sharply truncate at apex.

**Figure 3.**
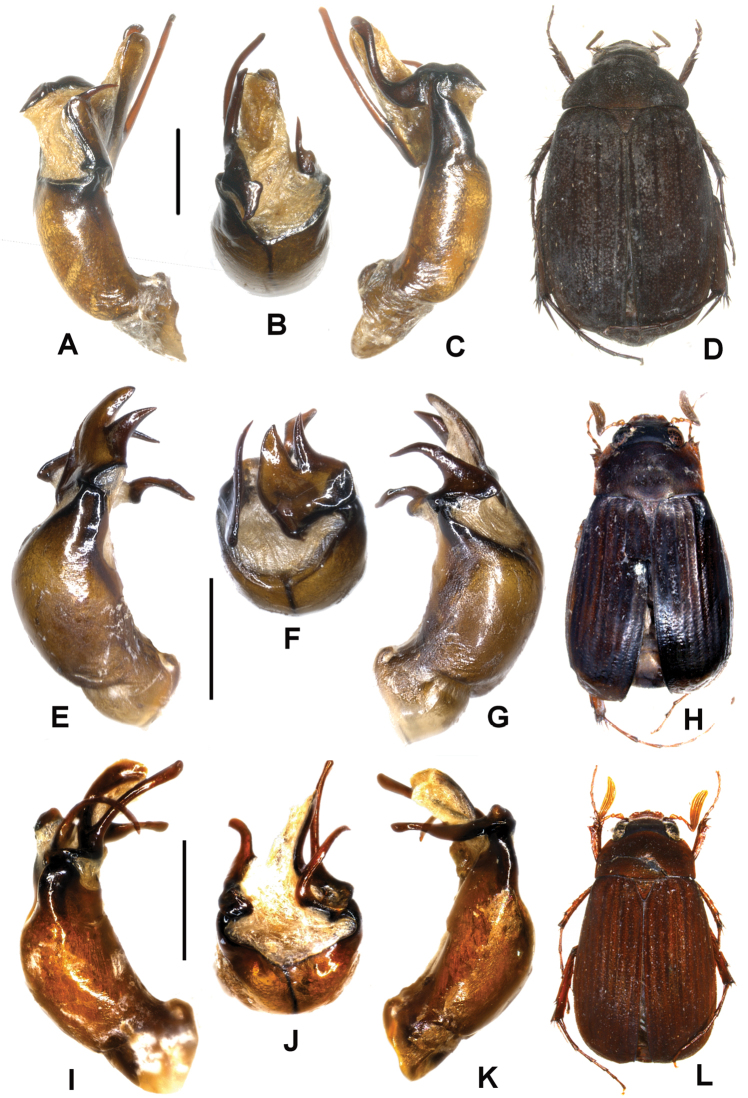
**A–D:** *Neoserica jiangxiensis* sp. n. (holotype) **E–H** *Neoserica kanphantensis* sp. n. (holotype) **I–L** *Neoserica trifida* sp. n. (holotype) **A, E, I** Aedeagus, left side lateral view **C, G, K** Aedeagus, right side lateral view **B, F, J** parameres, dorsal view **D, H, L** Habitus. Scale: 1 mm. Habitus not to scale.

Aedeagus: Fig. 3A–C. Female unknown.

#### Diagnosis.

*Neoserica jiangxiensis* is very similar to *Neoserica euyunnanica* externally and in the general shape of male genitalia. It differs by the left paramere being deeply subdivided into three lobes, the dorsal one is more than half as long as the median one and has a strong hook directed ventrally; the dorsal lobe of the right paramere is slightly shorter and basally narrower than in *Neoserica euyunnanica*.

#### Variation.

Body length: 13.5–14.0 mm, length of elytra: 10.0–10.5 mm, width: 8.0–8.2 mm.

#### Etymology.

The new species is named “*jiangxiensis*” according to its occurrence in Jiangxi (China).

### Neoserica (s. l.) kanphantensis sp. n.

Taxon classificationAnimaliaColeopteraScarabaeidae

http://zoobank.org/B98BCC41-9654-4E6C-834E-1A2E69F84703

Figs 3E–H , 8

#### Type material examined.

Holotype ♂ “Myanmar (Burma), Province Kachin State Kanphant/ Grenze to China, 29–30.05.2006, N26°08'51.2', E, 098°34'58.2" Nachtfang Leg. M. Langer, S. Naumann, & S. Loeffler/ Coll. M. Langer” (ZFMK). Paratypes. 1 ♂ “Myanmar (Burma), Province Kachin State Kanphant/ Grenze to China, 29–30.05.2006, 26°08'51.2'N, 098°34'58.2"E Nachtfang Leg. M. Langer, S. Naumann, & S. Loeffler/ Coll. M. Langer” (ZFMK), 1 ♂ “Myanmar (Burma), Kachin State, Hpiman; 21.–23.V.2002; leg. A. Azuma” (ZFMK), 1 ♂ “[China] Yunnan, Pianma, 2011-V-10, N: 26.018 E: 98.625, 1970m” (IZAS).

#### Description.

Body length: 11 mm, length of elytra: 7.6 mm, width: 5.2 mm. Body oblong, dark brown, antennal club yellowish brown, anterior labroclypeus shiny, dorsal surface dull, nearly glabrous.

Labroclypeus subtrapezoidal, little wider than long, widest at base, lateral margins weakly convex and convergent anteriorly, anterior angles weakly rounded, anterior margin weakly sinuate medially, margins strongly reflexed; surface flat and shiny, basis without dull toment, punctation dense, behind the anterior margin with coarser punctures each bearing a long erect seta; frontoclypeal suture indistinctly incised, flat and distinctly curved medially; smooth area anterior to eye approximately twice as wide as long; ocular canthus long and slender, glabrous, with a few minute and superficial punctures, with a long terminal seta. Frons dull, with fine and sparse punctures, beside the eyes and behind the frontoclypeal suture with a few erect setae. Eyes large, ratio diameter/interocular width: 0.69. Antenna with ten antennomeres, club with six antennomeres, moderately reflexed, twice as long as remaining antennomeres combined; antennomere 5 subequal to length of club, antennomere 4 strongly transverse, antennomere 3 half as long as pedicellus. Mentum elevated and slightly flattened anteriorly. Labrum weakly produced medially, with a moderate median sinuation.

Pronotum transverse, subrectangular, widest at base, lateral margins in basal half straight and nearly subparallel, evenly convex and strongly convergent in anterior half, anterior angles sharp and strongly produced, posterior angles right-angled, slightly rounded at the tip; anterior margin strongly convexly produced medially, marginal line incomplete medially; surface densely and finely punctate, with minute setae in punctures; setae of lateral border nearly absent; hypomeron basally distinctly carinate, but carina not produced. Scutellum moderately wide and short, with fine, moderately dense punctures, smooth on basal midline, with only minute setae.

Elytra oblong, widest shortly behind middle, striae weakly impressed, finely and moderately densely punctate, intervals weakly convex with punctures concentrated along the striae, odd intervals with a few fine, white, adpressed setae, otherwise only with very minute setae in punctures; epipleural edge fine, ending at the blunt external apical angle of elytra, epipleura densely setose, apical border narrowly membraneous, with a fine fringe of microtrichomes (visible at 100× magnification).

Ventral surface dull, coarsely and densely punctate, metasternum glabrous; metacoxa glabrous, with a few short setae laterally, posterior margin straight; abdominal sternites finely and unevenly and not densely punctuate, nearly glabrous, with a transverse row of coarse punctures, each bearing a robust short seta. Mesosternum between mesocoxae half as wide as slender mesofemur. Ratio of length of metepisternum/metacoxa: 1/1.47. Pygidium weakly convex and dull, finely and densely punctate, without a smooth midline, with a few longer setae on posterior half.

Legs slender; femora with two longitudinal rows of setae, finely and sparsely punctate between the rows; metafemur dull, anterior margin acute, behind anterior edge without serrated line, punctures and setae of anterior longitudinal row completely reduced, posterior margin in apical half ventrally smooth and not widened but slightly serrate in apical quarter, posterior margin dorsally distinctly serrated, on its basal portion with a few short setae. Metatibia slender and long, widest at apex, ratio of width/length: 1/4.3, sharply carinate dorsally, with two groups of spines, basal group at one third, apical group at three quarters of metatibial length, basally with a few robust but single setae; lateral face longitudinally convex, very finely, superficially and sparsely punctate, subdorsal longitudinal carina on lateral face present on about two third of metatibial length, but not very distinct; ventral edge finely serrated, with three robust setae of which the two distal ones are widely separated; medial face smooth, apex moderately distinctly concavely sinuate interiorly near tarsal articulation. Tarsomeres ventrally with sparse, short setae, laterally not carinate, protarsomeres smooth, meso- and metatarsomeres with a few fine punctures; metatarsomeres with a strongly serrated ridge ventrally and a sharp subventral carina immediately beside it, first metatarsomere distinctly longer than following two tarsomeres combined and distinctly longer than dorsal tibial spur. Protibia long, bidentate; anterior claws symmetrical, basal tooth of inner claw sharply truncate at apex.

Aedeagus: Fig. 3E–G. Female unknown.

#### Diagnosis.

The new species is in external morphology rather similar to *Neoserica trifida*. It differs (as from all other species of the *Neoserica abnormis* group) in the flattened and bifid left paramere (lateral view).

#### Variation.

Body length: 10.4–11.0 mm, length of elytra: 7.6–8.2 mm, width: 5.2–5.6 mm.

#### Etymology.

The new species is named after its type locality, Kanphant.

### Neoserica (s. l.) trifida sp. n.

Taxon classificationAnimaliaColeopteraScarabaeidae

http://zoobank.org/59A8C716-2B48-4A23-A8D1-D6FA7F1DD621

Figs 3I–L , 9

#### Type material examined.

Holotype: ♂ “China, Yunnan prov.; Gaoligongshan mts.; 90km W of Baoshan; S. Bečvář leg.; 26–29.v.1995/ Coll. P. Pacholátko Invt. No.” (CPPB). Paratypes: 5 ♂♂ “China, Yunnan prov.; Gaoligongshan mts.; 90km W of Baoshan; S. Bečvář leg.; 26–29.v.1995/ Coll. P. Pacholátko Invt. No.” (ZFMK), 32 ♂♂ “China, W Yunnan prov., mts. 60Km E Tengchong, 2300m 14.–19.v.2006, S. Murzin & I. Shokhin leg.” (CP, ZFMK).

#### Description.

Body length: 10.7 mm, length of elytra: 7.8 mm, width: 6.1 mm. Body oblong, dark brown, antennal club yellowish brown, anterior labroclypeus shiny, dorsal surface dull, nearly glabrous.

Labroclypeus subrectangular, little wider than long, widest at base, lateral margins straight and weakly convergent anteriorly, anterior angles moderately rounded, anterior margin weakly sinuate medially, margins strongly reflexed; surface flat and shiny, basis with dull toment, punctation dense, behind the anterior margin with coarser punctures each bearing a long erect seta; frontoclypeal suture indistinctly incised, flat and distinctly curved medially; smooth area anterior to eye approximately twice as wide as long; ocular canthus moderately long (length = 1/3 of ocular diameter) and slender, glabrous, with a few minute and superficial punctures, with a long terminal seta. Frons dull, in posterior half weakly shiny, with fine and sparse punctures, beside the eyes and behind the frontoclypeal suture with a few erect setae. Eyes large, ratio diameter/interocular width: 0.75. Antenna with ten antennomeres, club with six antennomeres, strongly reflexed, twice as long as remaining antennomeres combined; antennomere 5 subequal to half of length of club, antennomere 4 strongly transverse, antennomere 3 half as long as pedicellus. Mentum elevated and slightly flattened anteriorly. Labrum weakly produced medially, with a moderate median sinuation.

Pronotum transverse, subrectangular, widest at base, lateral margins in basal half straight and nearly subparallel, evenly convex and strongly convergent in anterior half, anterior angles moderately sharp and moderately produced, posterior angles blunt, slightly rounded at the tip; anterior margin strongly convexly produced medially, with a medially incomplete marginal line; surface densely and finely punctate, with minute setae in punctures; setae of lateral border fine and sparse; hypomeron basally distinctly carinate, but carina not produced. Scutellum moderately wide and long, with fine, moderately dense punctures, smooth on basal midline, with only minute setae.

Elytra oblong, widest in posterior third, striae weakly impressed, finely and moderately densely punctate, intervals weakly convex with punctures concentrated along the striae, odd intervals with a few fine, adpressed setae, otherwise only with very minute setae in punctures; epipleural edge fine, ending at the blunt external apical angle of elytra, epipleura densely setose, apical border narrowly membraneous, with a fine fringe of microtrichomes (visible at 100× magnification).

Ventral surface dull, coarsely and densely punctate, metasternum glabrous; metacoxa glabrous, with a few short setae laterally, posterior margin straight; abdominal sternites finely and unevenly and not densely punctuate, nearly glabrous, with a transverse row of coarse punctures, each bearing a robust short seta. Mesosternum between mesocoxae half as wide as slender mesofemur. Ratio of length of metepisternum/metacoxa: 1/1.4. Pygidium weakly convex and dull, finely and densely punctate, without a smooth midline, with a few longer setae on posterior half.

Legs slender; femora with two longitudinal rows of setae, finely and sparsely punctate between the rows; metafemur dull, anterior margin acute, behind anterior edge without serrated line, punctures and setae of anterior longitudinal row completely reduced, posterior margin in apical half ventrally smooth and not widened but slightly serrate in apical quarter, posterior margin dorsally distinctly serrated, on its basal portion with a few short setae. Metatibia slender and long, widest at apex, ratio of width/length: 1/5.1, sharply carinate dorsally, with two groups of spines, basal group just before the middle, apical group at three quarters of metatibial length, basally with a few robust but single setae; lateral face longitudinally convex, very finely, superficially and sparsely punctate, subdorsal longitudinal carina on lateral face present on about two third of metatibial length, but not very distinct; ventral edge finely serrated, with three robust setae of which the two distal ones are widely separated; medial face smooth, apex moderately distinctly concavely sinuate interiorly near tarsal articulation. Tarsomeres ventrally with sparse, short setae, laterally not carinate, protarsomeres smooth, meso- and metatarsomeres with a few fine punctures; metatarsomeres with a strongly serrated ridge ventrally and a sharp subventral carina immediately beside it, first metatarsomere as long as following two tarsomeres combined and slightly longer than dorsal tibial spur. Protibia long, bidentate; anterior claws symmetrical, basal tooth of inner claw sharply truncate at apex.

Aedeagus: Fig. 3I–K. Female unknown.

#### Diagnosis.

The new species differs from all other species of the *Neoserica abnormis* group by the convexly produced anterior margin of pronotum, the lacking scale-like setae on elytra and the serrate ventral posterior margin of metafemur.

#### Variation.

Body length: 10.5–10.7 mm, length of elytra: 7.5–7.8 mm, width: 5.9–6.1 mm.

#### Etymology.

The new species is named “*trifida*” with reference to its trifid left paramere.

### Neoserica (s. l.) yingjiangensis sp. n.

Taxon classificationAnimaliaColeopteraScarabaeidae

http://zoobank.org/41EFFEC9-1266-4FBE-89EA-4FE0FAF56744

Figs 4A–D , 9

#### Type material examined.

Holotype: ♂ “Yingjiang, Yunnan, 13.IV.1980, 1300m, leg. Li Hongxing” (IZAS). Paratypes: 1 ♂ “Yingjiang, Yunnan, 13.IV.1980, 1700m, leg. Gao Ping” (ZFMK), 1 ♂ “Yingjiang, Yunnan, 13.IV.1980, 300m, leg. Song Shimei” (IZAS).

#### Description.

Body length: 10.5 mm, length of elytra: 7.5 mm, width: 4.9 mm. Body oblong and slender, dark brown, antennal club yellowish brown, anterior labroclypeus shiny, dorsal surface dull, nearly glabrous.

Labroclypeus subtrapezoidal, distinctly wider than long, widest at base, lateral margins straight and moderately convergent anteriorly, anterior angles moderately rounded, anterior margin weakly sinuate medially, margins strongly reflexed; surface flat and shiny, basis with dull toment, punctation dense, behind anterior margin with a transversal row of coarser punctures each bearing a long erect seta; frontoclypeal suture indistinctly incised, flat and distinctly curved medially; smooth area anterior to eye approximately twice as wide as long; ocular canthus moderately long (length = 1/3 of ocular diameter) and slender, glabrous, with a few minute and superficial punctures and a long terminal seta. Frons dull, with fine and sparse punctures, beside the eyes with a few erect setae. Eyes large, ratio diameter/interocular width: 0.92. Antenna with ten antennomeres, club with six antennomeres, strongly reflexed, twice as long as remaining antennomeres combined; antennomere 5 subequal to length of club, antennomere 4 strongly transverse, antennomere 3 half as long as pedicellus. Mentum elevated and slightly flattened anteriorly. Labrum weakly produced medially, with a moderate median sinuation.

Pronotum transverse, subrectangular, widest at base, lateral margins in basal half straight and nearly subparallel and slightly concavely sinuate, evenly convex and strongly convergent in anterior half, anterior angles sharp and distinctly produced, posterior angles right-angled, slightly rounded at the tip; anterior margin strongly convexly produced medially, with a medially incomplete marginal line; surface densely and finely punctate, with minute setae in punctures; setae of lateral border fine and sparse; hypomeron basally distinctly carinate, but carina not produced. Scutellum moderately wide and long, with fine, moderately dense punctures, smooth on basal midline, with only minute setae.

Elytra oblong and very slender, widest just before posterior third, striae weakly impressed, finely and moderately densely punctate, intervals weakly convex with punctures concentrated along the striae, odd intervals with a few fine, adpressed setae, otherwise only with very minute setae in punctures; epipleural edge fine, ending at the blunt external apical angle of elytra, epipleura densely setose, apical border narrowly membraneous, with a fine fringe of microtrichomes (visible at 100× magnification).

Ventral surface dull, coarsely and densely punctate, metasternum glabrous; metacoxa glabrous, with a few short setae laterally, posterior margin straight; abdominal sternites finely and unevenly and not densely punctuate, nearly glabrous, with a transverse row of coarse punctures, each bearing a robust short seta. Mesosternum between mesocoxae half as wide as slender mesofemur. Ratio of length of metepisternum/metacoxa: 1/1.38. Pygidium weakly convex and dull, finely and densely punctate, without a smooth midline, with a few longer setae on posterior half.

Legs slender; femora with two longitudinal rows of setae, finely and sparsely punctate between the rows; metafemur dull, anterior margin acute, behind anterior edge without serrated line, punctures and setae of anterior longitudinal row completely reduced, posterior margin in apical half ventrally smooth and not widened but slightly serrate in apical quarter, posterior margin dorsally distinctly serrated, on its basal portion with a few short setae. Metatibia slender and long, widest at apex, ratio of width/length: 1/4.7, sharply carinate dorsally, with two groups of spines, basal group just before the middle, apical group at three quarters of metatibial length, basally with a few robust but single setae; lateral face longitudinally convex, very finely, superficially and sparsely punctate, subdorsal longitudinal carina on lateral face present on about two third of metatibial length, but nearly longitudinally convex and very indistinct; ventral edge finely serrated, with three robust setae of which the two distal ones are widely separated; medial face smooth, apex moderately distinctly concavely sinuate interiorly near tarsal articulation. Tarsomeres ventrally with sparse, short setae, laterally not carinate, protarsomeres smooth, meso- and metatarsomeres with a few fine punctures; metatarsomeres with a strongly serrated ridge ventrally and a sharp subventral carina immediately beside it, first metatarsomere as long as following two tarsomeres combined and slightly longer than dorsal tibial spur. Protibia long, bidentate; anterior claws symmetrical, basal tooth of inner claw sharply truncate at apex.

**Figure 4.**
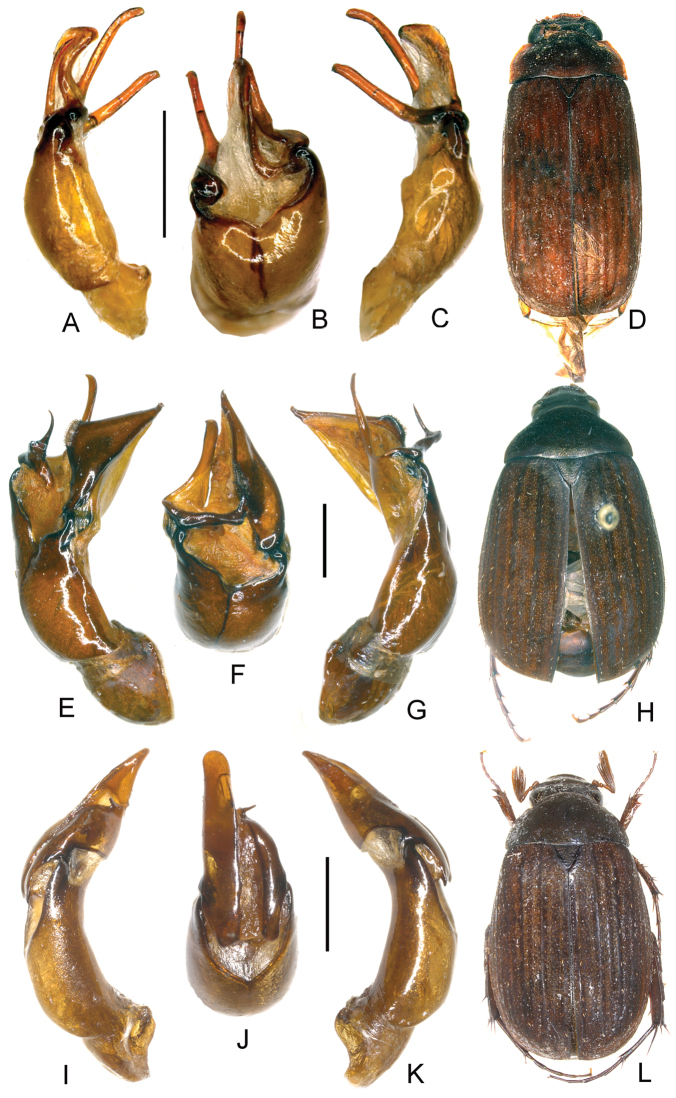
**A–D** *Neoserica yingjiangensis* sp. n. (holotype) **E–H** *Neoserica thailandensis* sp. n. (holotype) **I–L** *Neoserica lamellosa* sp. n. (holotype) **A, E, I** Aedeagus, left side lateral view **C, G, K** Aedeagus, right side lateral view **B, F, J** parameres, dorsal view **D, H, L** Habitus. Scale: 1 mm. Habitus not to scale.

Aedeagus: Fig. 4A–C. Female unknown.

#### Diagnosis.

The new species differs from *Neoserica trifida* in the more slender body, the straight dorsal lobe of the left paramere, the slightly shorter median lobe, as well as the larger eyes.

#### Variation.

Body length: 10.5–11.0 mm, length of elytra: 7.5–7.9 mm, width: 4.9–5.0 mm.

#### Etymology.

The new species is named after its type locality, Yingjiang.

### Neoserica (s. l.) thailandensis sp. n.

Taxon classificationAnimaliaColeopteraScarabaeidae

http://zoobank.org/8B12B98E-0A20-4256-9136-74802A14F362

Figs 4E–H , 8

#### Type material examined.

Holotype: ♂ “N-Thailand 25.-29.5.1990 Doi Inthanon leg. Malicky” (ZSMC). Paratype. 1 ♂ “N-Thailand 27.III.1990 Doi Inthang [sic, = Doi Inthanon] lg. Malicky” (ZFMK), 1 ♂ “N. Thailand Doi Pui, near Chiang Mai; 09.V.1985; leg. H. Nara” (ZFMK).

#### Description.

Body length: 14.5 mm, length of elytra: 10.7 mm, width: 8.3 mm. Body oblong, dark brown, antennal club yellowish brown, anterior labroclypeus shiny, dorsal surface dull, sparsely setose.

Labroclypeus subtrapezoidal, distinctly wider than long, widest at base, lateral margins weakly convex and strongly convergent anteriorly, anterior angles moderately rounded, anterior margin weakly sinuate medially, margins moderately reflexed; surface nearly flat and shiny, basis with dull toment, punctation dense, anteriorly more sparse, behind the anterior margin with coarse punctures each bearing a long erect seta; frontoclypeal suture indistinctly incised, flat and distinctly curved medially; smooth area anterior to eye approximately 1.5 times as wide as long; ocular canthus moderately long (length = 1/3 of ocular diameter) and slender, glabrous, with a long terminal seta. Frons dull, with fine and sparse punctures, beside the eyes with a few erect setae. Eyes small, ratio diameter/interocular width: 0.48. Antenna with ten antennomeres, club with six antennomeres, straight, as long as remaining antennomeres combined; antennomere 5 subequal to half of length of club, antennomere 4 strongly transverse, antennomere 3 half as long as pedicellus. Mentum elevated and slightly flattened anteriorly. Labrum distinctly produced medially, with a moderate median sinuation.

Pronotum moderately transverse, subtrapezoidal, widest at base, lateral margins evenly convex and moderately convergent anteriorly, in anterior half more strongly convergent, anterior angles sharp and distinctly produced, posterior angles blunt, slightly rounded at the tip; anterior margin nearly straight, with a distinct and complete marginal line; surface densely and finely punctate with minute setae in punctures; setae of lateral border sparse; hypomeron basally distinctly carinate, but carina only weakly produced. Scutellum moderately wide and long, triangular with nearly straight sides, apex slightly rounded, with fine, dense punctures, smooth along the middle, with only minute setae.

Elytra oblong, apex slightly truncate and slightly concavely sinuated before the apical angle, widest shortly behind the middle, striae weakly impressed, finely and moderately densely punctate, odd intervals slightly convex with punctures concentrated along the striae, others evenly punctate and nearly flat, odd intervals with white scale-like, adpressed setae, otherwise only with very minute setae in punctures; epipleural edge fine, very narrow behind the middle, ending at the blunt external apical angle of elytra, epipleura only sparsely setose, apical border narrowly membraneous, with only a very fine fringe of microtrichomes (visible at 100× magnification).

Ventral surface dull, coarsely and densely punctate, metasternum glabrous; metacoxa glabrous, with a few short setae laterally, posterior margin straight; abdominal sternites finely and unevenly densely punctuate, nearly glabrous, with a transverse row of coarse punctures, each bearing a robust short seta. Mesosternum between mesocoxae half as wide as slender mesofemur. Ratio of length of metepisternum/metacoxa: 1/1.54. Pygidium weakly convex and dull, coarsely and densely punctate, with a very narrow smooth midline, with a few longer setae on sides and along the apical margin, otherwise only with minute setae in punctures.

Legs slender; femora with two longitudinal rows of setae, finely and sparsely punctate between the rows; metafemur dull, anterior margin acute, behind anterior edge without serrated line, setae of anterior longitudinal row with very sparse and short setae only, posterior margin in apical half ventrally smooth and slightly widened, posterior margin dorsally distinctly serrated, on its basal portion with a few short setae. Metatibia slender and moderately long, widest at apex, ratio of width/length: 1/4.0, sharply carinate dorsally, with two groups of spines, basal group just before the middle, apical group at three quarters of metatibial length, basally with a few robust but single setae; lateral face longitudinally convex, very finely, superficially and sparsely punctate, subdorsal longitudinal carina on lateral face present on about two third of metatibial length; ventral edge finely serrated, with four robust equidistant setae; medial face smooth, apex moderately concavely sinuate interiorly near tarsal articulation. Tarsomeres ventrally with sparse, short setae, laterally not carinate, protarsomeres smooth, meso- and metatarsomeres with a few very fine punctures; metatarsomeres ventrally glabrous, with a strongly serrated ridge ventrally and a sharp subventral carina immediately beside it, first metatarsomere slightly longer than following two tarsomeres combined and slightly longer than dorsal tibial spur. Protibia long, bidentate; anterior claws symmetrical, basal tooth of inner claw sharply truncate at apex.

Aedeagus: Fig. 4E–G. Female unknown.

#### Diagnosis.

*Neoserica thailandensis* sp. n. differs from all other species of the *Neoserica abnormis* group by the apex of elytra being slightly concave before the apical angle; furthermore, it differs in shape of parameres: the left paramere is triangular and robust not being subdivided in lobes.

#### Variation.

Body length: 12.9–14.5 mm, length of elytra: 9.9–10.7 mm, width: 7.8–8.3 mm.

#### Etymology.

The new species is named “*thailandensis*” with reference to its occurrence in Thailand.

### Neoserica (s. l.) lamellosa sp. n.

Taxon classificationAnimaliaColeopteraScarabaeidae

http://zoobank.org/B7AD7304-5B7B-477C-866F-09FD51EB5391

Figs 4I–L , 9

#### Type material examined.

Holotype: ♂ “N-Vietnam Tam Dao, Vinh Phu Prov. 21°27'18"N, 105°38'58"E 1050–1200m 2.–6.VI.1999 leg. Fabrizi, Jäger, Ahrens” (ZFMK). Paratypes: 1 ♀ “N-Vietnam Tam Dao, Vinh Phu Prov. 21°27'18"N, 105°38'58"E 1050–1200m 2.–6.VI.1999 leg. Fabrizi, Jäger, Ahrens” (ZFMK), 1 ♂ “Vietnam N. 15.5.–16.6. 75 km NW from Hanoi Tam Dao E. Jendek leg.” (CA), 1 ♂ “N. Vietnam/ Tonkin/ Tamdao/ pr. Vinhphu/ 2.–11.6.1985 Vit Kubáň leg./ VS 50” (CPPB).

#### Description.

Body length: 10.6 mm, length of elytra: 7.8 mm, width: 6.1 mm. Body oblong, dark brown, antennal club yellowish brown, anterior labroclypeus shiny, dorsal surface dull, opaque toment on elytra and pronotum less thick, with a light trace of shine, sparsely setose.

Labroclypeus slightly subrectangular, distinctly wider than long, widest at base, lateral margins nearly straight and weakly convergent anteriorly, anterior angles strongly rounded, anterior margin weakly sinuate medially, margins moderately reflexed; surface convexly elevated at middle and shiny, basis with dull toment, punctation moderately dense, anteriorly more sparse, behind the anterior margin with coarse punctures each bearing a long erect seta; frontoclypeal suture distinctly incised, flat and distinctly curved medially; smooth area anterior to eye approximately 1.5 times as wide as long; ocular canthus moderately wide and long (length = 1/3 of ocular diameter), glabrous, with a fine terminal seta. Frons dull, with fine and dense punctures, beside the eyes a with a few erect setae. Eyes small, ratio diameter/interocular width: 0.48. Antenna with ten antennomeres, club with seven antennomeres, straight, nearly twice as long as remaining antennomeres combined; antennomere 5 equal to length of club, antennomere 4 subequal to three quarter of length of club, antennomere 3 half as long as pedicellus. Mentum elevated and slightly flattened anteriorly. Labrum distinctly produced medially, with a moderate median sinuation.

Pronotum moderately transverse, subtrapezoidal, widest at base, lateral margins evenly convex and convergent anteriorly, anterior angles sharp and distinctly produced, posterior angles blunt, strongly rounded at the tip; anterior margin nearly straight, with a fine and complete marginal line; surface densely and finely punctate with minute setae in punctures; setae of lateral border sparse; hypomeron basally distinctly carinate, but carina only weakly produced. Scutellum long, triangular with nearly straight sides, apex slightly rounded, with fine, dense punctures, with only minute setae.

Elytra oblong, widest shortly behind the middle, striae weakly impressed, finely and moderately densely punctate, odd intervals distinctly convex with punctures concentrated along the striae, others evenly punctate and nearly flat, odd intervals with white scale-like, adpressed setae, otherwise only with very minute setae in punctures; epipleural edge fine, very narrow behind the middle, ending at the moderately rounded external apical angle of elytra, epipleura densely setose, apical border chitinous, with nearly invisible fringe of microtrichomes (visible at 100× magnification).

Ventral surface dull, coarsely and densely punctate, metasternum sparsely covered with setae on the disc, glabrous on sides; metacoxa glabrous, with a few short setae laterally, posterior margin weakly convex; abdominal sternites finely and unevenly densely punctuate, nearly glabrous, with a transverse row of coarse punctures, each bearing a robust short seta. Mesosternum between mesocoxae half as wide as slender mesofemur. Ratio of length of metepisternum/metacoxa: 1/1.77. Pygidium weakly convex and dull, densely punctate, fine punctures mixed with coarser ones, with a narrow smooth midline, nearly glabrous, only with minute setae in punctures.

Legs slender; femora with two longitudinal rows of setae, finely and sparsely punctate between the rows; metafemur dull, anterior margin acute, behind anterior edge without serrated line, setae of anterior longitudinal row nearly completely lacking, posterior margin in apical half ventrally smooth and slightly widened, posterior margin dorsally distinctly serrated, on its basal portion with a few short setae. Metatibia moderately slender and long, widest at apex, ratio of width/length: 1/3.7, sharply carinate dorsally, with two groups of spines, basal group just before the middle, apical group at three quarters of metatibial length, basally with a few robust but single setae; lateral face longitudinally convex, very finely, superficially and sparsely punctate, subdorsal longitudinal carina on lateral face present on about two third of metatibial length; ventral edge finely serrated, with three robust equidistant setae; medial face smooth, apex moderately concavely sinuate interiorly near tarsal articulation. Tarsomeres ventrally with sparse, short setae, laterally not carinate, protarsomeres smooth, meso- and metatarsomeres with a few very fine punctures; metatarsomeres ventrally glabrous, with a strongly serrated ridge ventrally and a sharp subventral carina immediately beside it, first metatarsomere as long as following two tarsomeres combined and slightly longer than dorsal tibial spur. Protibia long, bidentate; anterior claws symmetrical, basal tooth of inner claw sharply truncate at apex.

Aedeagus: Fig. 4I–K.

#### Diagnosis.

*Neoserica lamellosa* sp. n. differs from *Neoserica cardamomensis* and *Neoserica namthaensis* in the slightly longer antennal club and the shape of parameres being not subdivided into separate lobes but being robust and compact.

#### Variation.

Body length: 10.25–13.6 mm, length of elytra: 7.8–9.6 mm, width: 6.1–7.1 mm. Female: Antennal club composed of four antennomeres, as long as remaining antennomeres combined; pygidium less convex.

#### Etymology.

The new species is named “*lamellosa*” with reference to its antennal club in males composed of many (seven) lamellae.

### Neoserica (s. l.) putaoana sp. n.

Taxon classificationAnimaliaColeopteraScarabaeidae

http://zoobank.org/37411B0C-A144-46DA-937E-EC3154D61F05

Figs 5A–D , 8

#### Type material examined.

Holotype: ♂ “Myanmar N (Burma) 25km E Putao, H-800m Nan Sa Bon vill. 06–09.05.1998 leg. S. Murzin & V. Siniaev/ Coll. Takeshi Itoh Osaka (Japan)” (ZFMK). Paratype: 1 ♀ “Myanmar N (Burma) 25km E Putao, H-800m Nan Sa Bon vill. 06–09.05.1998 leg. S. Murzin & V. Siniaev/ Coll. Takeshi Itoh Osaka (Japan)” (ZFMK).

#### Description.

Body length: 13.5 mm, length of elytra: 10.0 mm, width: 8.3 mm. Body oblong, dark brown, antennal club yellowish brown, anterior labroclypeus shiny, dorsal surface dull, sparsely setose.

Labroclypeus subtrapezoidal, distinctly wider than long, widest at base, lateral margins moderately convex and convergent anteriorly, anterior angles strongly rounded, anterior margin weakly sinuate medially, margins moderately reflexed; surface nearly flat and shiny, basis with dull toment, punctation dense, anteriorly more sparse, behind the anterior margin with coarse punctures each bearing a long erect seta; frontoclypeal suture indistinctly incised, flat and distinctly curved medially; smooth area anterior to eye approximately 1.5 times as wide as long; ocular canthus moderately long (length = 1/3 of ocular diameter) and slender, glabrous, with a long terminal seta. Frons dull, with fine and sparse punctures, beside the eyes with a few erect setae. Eyes small, ratio diameter/interocular width: 0.5. Antenna with ten antennomeres, club with six antennomeres, straight, only slightly longer than the remaining antennomeres combined; antennomere 5 subequal to two thirds of length of club, antennomere 4 slightly transverse, antennomere 3 half as long as pedicellus. Mentum elevated and slightly flattened anteriorly. Labrum distinctly produced medially, with a moderate median sinuation.

Pronotum moderately transverse, subtrapezoidal, widest at base, lateral margins straight in the basal half and slightly weakly convergent anteriorly, convex and strongly convergent in anterior half, anterior angles sharp and distinctly produced, posterior angles blunt, slightly rounded at the tip; anterior margin nearly straight, with a distinct and complete marginal line; surface densely and finely punctate with minute setae in punctures; setae of anterior and lateral border sparse; hypomeron basally distinctly carinate, but carina only weakly produced. Scutellum wide and moderately long, triangular with nearly straight sides, apex slightly rounded, with fine, moderately dense punctures, basally smooth at middle, with only minute setae.

Elytra oblong, apex slightly truncate, widest shortly behind the middle, striae weakly impressed, finely and moderately densely punctate, odd intervals slightly convex with punctures concentrated along the striae, others evenly punctate and nearly flat, odd intervals with white scale-like, adpressed setae, otherwise only with very minute setae in punctures; epipleural edge fine, very narrow behind the middle, ending at the blunt external apical angle of elytra, epipleura only sparsely setose, apical border narrowly membraneous, with only a very fine fringe of microtrichomes (visible at 100× magnification).

Ventral surface dull, coarsely and densely punctate, metasternum glabrous; metacoxa glabrous, with a few short setae laterally, posterior margin weakly convex; abdominal sternites finely and unevenly densely punctuate, nearly glabrous, with a transverse row of coarse punctures, each bearing a robust short seta. Mesosternum between mesocoxae half as wide as slender mesofemur. Ratio of length of metepisternum/metacoxa: 1/1.73. Pygidium weakly convex and dull, coarsely and densely punctate, without smooth midline, with a few semi-erect setae basally on sides and along the apical margin.

Legs slender; femora with two longitudinal rows of setae, finely and sparsely punctate between the rows; metafemur dull, anterior margin acute, behind anterior edge without serrated line, setae of anterior longitudinal row nearly completely lacking, posterior margin in apical half ventrally smooth and slightly widened, posterior margin dorsally distinctly serrated, on its basal portion with a few short setae. Metatibia slender and moderately long, widest at apex, ratio of width/length: 1/3.4, sharply carinate dorsally, with two groups of spines, basal group just before the middle, apical group at three quarters of metatibial length, basally with a few robust but single setae; lateral face longitudinally convex, very finely, superficially and sparsely punctate, subdorsal longitudinal carina on lateral face present on about two third of metatibial length; ventral edge finely serrated, with four robust equidistant setae; medial face smooth, apex moderately concavely sinuate interiorly near tarsal articulation. Tarsomeres ventrally with sparse, short setae, laterally not carinate, protarsomeres smooth, meso- and metatarsomeres with a few very fine punctures; metatarsomeres ventrally glabrous, with a strongly serrated ridge ventrally and a sharp subventral carina immediately beside it, first metatarsomere slightly longer than following two tarsomeres combined and slightly longer than dorsal tibial spur. Protibia long, bidentate; anterior claws symmetrical, basal tooth of inner claw sharply truncate at apex.

**Figure 5.**
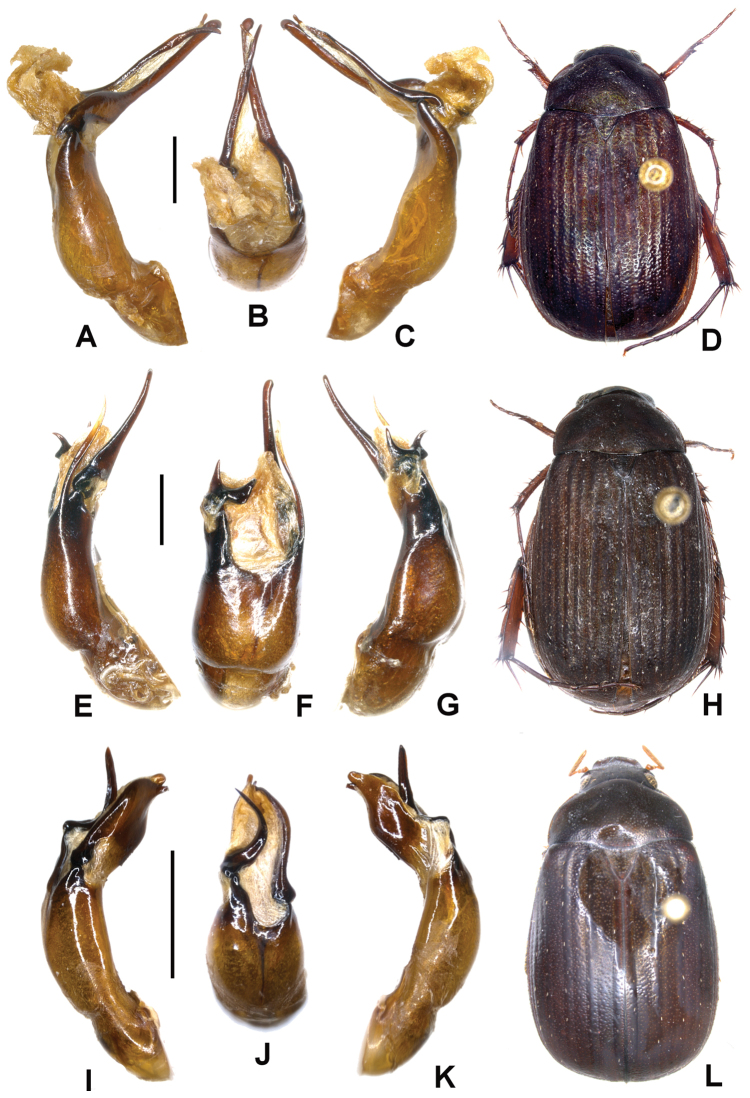
**A–D** *Neoserica putaoana* sp. n. (holotype) **E–H** *Neoserica alloputaoana* sp. n. (holotype) **I–L** *Neoserica simplicissima* sp. n. (holotype) **A, E, I** Aedeagus, left side lateral view **C, G, K** Aedeagus, right side lateral view **B, F, J** parameres, dorsal view **D, H, L** Habitus. Scale: 1 mm. Habitus not to scale.

Aedeagus: Fig. 5A–D.

#### Diagnosis.

*Neoserica putaoana* sp. n. differs from *Neoserica abnormis* and related species by the narrow, long and slender parameres.

#### Variation.

Female: Club with four antennomeres, 6^th^ antennomere distinctly transverse but much shorter than the club.

#### Etymology.

The new species is named according its type locality Putao.

### Neoserica (s. l.) alloputaoana sp. n.

Taxon classificationAnimaliaColeopteraScarabaeidae

http://zoobank.org/A496E9B8-2781-48B3-B57A-49736BA3499D

Figs 5E–H , 9

#### Type material examined.

Holotype: ♂ “Myanmar N (Burma) 25km E Putao, H-800m Nan Sa Bon vill. 06–09.05.1998 leg. S. Murzin & V. Siniaev” (ZFMK). Paratypes: 1 ♀ “Myanmar N (Burma) 25km E Putao, H-800m Nan Sa Bon vill. 06-09.05.1998 leg. S. Murzin & V. Siniaev” (ZFMK), 4 ♂♂ “Myanmar N (Burma) 65 km NE Putao, 1250 m Zi Yar Dam vill. 18/21.05.1998 leg. S. Murzin & V. Sinaev” (ZFMK).

#### Description.

Body length: 13.0 mm, length of elytra: 9.9 mm, width: 7.7 mm. Body oblong, dark brown, antennal club yellowish brown, anterior labroclypeus shiny, dorsal surface dull, sparsely setose.

Labroclypeus subtrapezoidal, distinctly wider than long, widest at base, lateral margins moderately convex and convergent anteriorly, anterior angles strongly rounded, anterior margin weakly sinuate medially, margins moderately reflexed; surface nearly flat and shiny, basis with dull toment, punctation dense, anteriorly more sparse, behind the anterior margin with coarse punctures each bearing a long erect seta; frontoclypeal suture indistinctly incised, flat and distinctly curved medially; smooth area anterior to eye approximately 1.5 times as wide as long; ocular canthus moderately long (length = 1/3 of ocular diameter) and slender, glabrous, with a long terminal seta. Frons dull, with fine and sparse punctures, beside the eyes with a few erect setae. Eyes small, ratio diameter/interocular width: 0.64. Antenna with ten antennomeres, club with six antennomeres, straight, only slightly longer than the remaining antennomeres combined; antennomere 5 subequal to length of club, antennomere 4 strongly transverse, antennomere 3 half as long as pedicellus. Mentum elevated and slightly flattened anteriorly. Labrum distinctly produced medially, with a moderate median sinuation.

Pronotum moderately transverse, subtrapezoidal, widest at base, lateral margins evenly convex and moderately convergent anteriorly, in anterior half more strongly convergent, anterior angles sharp and distinctly produced, posterior angles blunt, slightly rounded at the tip; anterior margin nearly straight, with a distinct and complete marginal line; surface densely and finely punctate with minute setae in punctures; setae of lateral border sparse; hypomeron basally distinctly carinate, but carina only weakly produced. Scutellum moderately wide and long, triangular with nearly straight sides, apex slightly rounded, with fine, moderately dense punctures, smooth along the middle, with only minute setae.

Elytra oblong, apex slightly truncate, widest shortly behind the middle, striae weakly impressed, finely and moderately densely punctate, odd intervals slightly convex with punctures concentrated along the striae, others evenly punctate and nearly flat, odd intervals with white scale-like, adpressed setae, otherwise only with very minute setae in punctures; epipleural edge fine, very narrow behind the middle, ending at the blunt external apical angle of elytra, epipleura only sparsely setose, apical border narrowly membraneous, with only a very fine fringe of microtrichomes (visible at 100× magnification).

Ventral surface dull, coarsely and densely punctate, metasternum glabrous; metacoxa glabrous, with a few short setae laterally, posterior margin weakly convex; abdominal sternites finely and unevenly densely punctuate, nearly glabrous, with a transverse row of coarse punctures, each bearing a robust short seta. Mesosternum between mesocoxae half as wide as slender mesofemur. Ratio of length of metepisternum/metacoxa: 1/1.67. Pygidium weakly convex and dull, coarsely and densely punctate, without smooth midline, with a few short setae on sides and along the apical margin.

Legs slender; femora with two longitudinal rows of setae, finely and sparsely punctate between the rows; metafemur dull, anterior margin acute, behind anterior edge without serrated line, setae of anterior longitudinal row nearly completely lacking, posterior margin in apical half ventrally smooth and slightly widened, posterior margin dorsally distinctly serrated, on its basal portion with a few short setae. Metatibia slender and moderately long, widest at apex, ratio of width/length: 1/3.9, sharply carinate dorsally, with two groups of spines, basal group just before the middle, apical group at three quarters of metatibial length, basally with a few robust but single setae; lateral face longitudinally convex, very finely, superficially and sparsely punctate, subdorsal longitudinal carina on lateral face present on about two third of metatibial length; ventral edge finely serrated, with four robust equidistant setae; medial face smooth, apex moderately concavely sinuate interiorly near tarsal articulation. Tarsomeres ventrally with sparse, short setae, laterally not carinate, protarsomeres smooth, meso- and metatarsomeres with a few very fine punctures; metatarsomeres ventrally glabrous, with a strongly serrated ridge ventrally and a sharp subventral carina immediately beside it, first metatarsomere slightly longer than following two tarsomeres combined and slightly longer than dorsal tibial spur. Protibia long, bidentate; anterior claws symmetrical, basal tooth of inner claw sharply truncate at apex.

Aedeagus: Fig. 5E–G.

#### Diagnosis.

*Neoserica alloputaoana* sp. n. differs from *Neoserica putaoana* by the extremely short right paramere and the long lateral process of the right apical phallobase.

#### Variation.

Female: antennal club with four antennomeres, 6^th^ antennomere distinctly transverse but distinctly shorter than club.

#### Etymology.

The name of the new species is composed of the Greek prefix allo- and “*putaoana*” underlining it distinctiveness from the syntopically co-occurring *Neoserica putaoana*.

### Neoserica (s. l.) simplicissima sp. n.

Taxon classificationAnimaliaColeopteraScarabaeidae

http://zoobank.org/347436CF-68CC-4DE0-895E-55D9726DBF29

Figs 5I–L , 9

#### Type material examined.

Holotype ♂ “Laos-NE Hua Phan prov., 20°12'N, 104°01'E, Phu Phan Mt., 1500-1900m, 17.5.-3.6.2007, leg. C. Holzschuh” (ZFMK). Paratypes: 2 ♂♂ “Laos-NE Hua Phan prov., 20°12'N, 104°01'E, Phu Phan Mt., 1500-1900m, 17.5.-3.6.2007, leg. Vit Kubáň” (NMPC), 1 ♂, 1 ♀ “Laos-NE Hua Phan prov., 20°12'N, 104°01'E, Phu Phan Mt., 1500-1900m, 17.5.-3.6.2007, leg. C. Holzschuh” (ZFMK).

#### Description.

Body length: 12.2 mm, length of elytra: 8.8 mm, width: 7.3 mm. Body oblong, dark brown, antennal club brown, anterior labroclypeus shiny, dorsal surface dull, partially dull toment lost and moderately shiny, sparsely setose.

Labroclypeus subtrapezoidal, distinctly wider than long, widest at base, lateral margins moderately convex and convergent anteriorly, anterior angles moderately rounded, anterior margin weakly sinuate medially, margins moderately reflexed; surface nearly flat and shiny, basis with dull toment, punctation dense, anteriorly more sparse, behind the anterior margin with a transverse row of coarse punctures each bearing a long erect seta; frontoclypeal suture indistinctly incised, flat and distinctly curved medially; smooth area anterior to eye approximately 1.5 times as wide as long; ocular canthus moderately long (length = 1/3 of ocular diameter) and slender, glabrous, with a fine terminal seta. Frons dull, with fine and sparse punctures, beside eyes with a few erect setae. Eyes small, ratio diameter/interocular width: 0.53. Antenna with ten antennomeres, club with six antennomeres, straight, as long as remaining antennomeres combined; antennomere 5 half as long as the club, antennomere 6 three quarters as long as club, antennomere 4 slightly transverse, antennomere 3 half as long as pedicellus. Mentum elevated and slightly flattened anteriorly. Labrum distinctly produced medially, with a moderate median sinuation.

Pronotum moderately transverse, subtrapezoidal, widest at base, lateral margins evenly convex and strongly convergent anteriorly, anterior angles sharp and distinctly produced, posterior angles blunt, slightly rounded at the tip; anterior margin nearly straight, with a distinct and complete marginal line; surface densely and finely punctate with minute setae in punctures; setae of anterior and lateral border sparse; hypomeron basally distinctly carinate, but carina only weakly produced. Scutellum moderately long, triangular with convex sides and with the apex slightly rounded, with fine, moderately dense punctures, with only minute setae.

Elytra oblong, widest shortly behind middle, striae weakly impressed, finely and moderately densely punctate, intervals nearly flat, with moderately dense evenly spaced, fine punctures, intervals with a few fine white setae, otherwise only with very minute setae in punctures; epipleural edge fine, ending at the blunt external apical angle of elytra, epipleura sparsely setose, apical border chitinous, with only a very fine fringe of microtrichomes (visible at 100× magnification).

Ventral surface dull, coarsely and densely punctate, metasternum sparsely covered with setae on the disc, glabrous on sides; metacoxa glabrous, with a few short setae laterally, posterior margin weakly convex; abdominal sternites finely and unevenly densely punctuate, nearly glabrous, with a transverse row of coarse punctures, each bearing a robust short seta. Mesosternum between mesocoxae half as wide as slender mesofemur. Ratio of length of metepisternum/metacoxa: 1/1.59. Pygidium weakly convex and dull, coarsely and densely punctate, without smooth midline, with a few semi-erect setae beside the apical margin.

Legs slender; femora with two longitudinal rows of setae, finely and sparsely punctate between the rows; metafemur dull, anterior margin acute, behind anterior edge without serrated line, setae of anterior longitudinal row nearly completely lacking, posterior margin in apical half ventrally smooth and slightly widened, posterior margin dorsally distinctly serrated, on its basal portion with a few short setae. Metatibia slender and long, widest at apex, ratio of width/length: 1/4.0, sharply carinate dorsally, with two groups of spines, basal group just before the middle, apical group at three quarters of metatibial length, basally with a few robust but single setae; lateral face longitudinally convex, very finely, superficially and sparsely punctate, subdorsal longitudinal carina on lateral face present on about two third of metatibial length; ventral edge finely serrated, with three robust equidistant setae; medial face smooth, apex moderately concavely sinuate interiorly near tarsal articulation. Tarsomeres ventrally with sparse, short setae, laterally not carinate, protarsomeres smooth, meso- and metatarsomeres with a few very fine punctures; metatarsomeres ventrally glabrous, with a strongly serrated ridge ventrally and a sharp subventral carina immediately beside it, first metatarsomere slightly longer than following two tarsomeres combined and slightly longer than dorsal tibial spur. Protibia long, bidentate; anterior claws symmetrical, basal tooth of inner claw sharply truncate at apex.

Aedeagus: Fig. 5I–K.

#### Diagnosis.

*Neoserica simplicissima* differs from all species with an antennal club composed of six antennomeres and a non-elongate left paramere in the absence of the basal process of the right paramere.

#### Variation.

Body length: 12.2–12.8 mm, length of elytra: 8.8–9.6 mm, width: 7.3–8.2 mm. Female: antennal club composed of fives antennomeres, slightly shorter than remaining antennomeres combined, 6^th^ antennomere one third as long as club, 7^th^ one slightly transversely produced.

#### Etymology.

The new species is named “*simplicissima*” (Latin adjective, meaning “very simple”) with reference to the lacking basal lobe of the right paramere.

### Neoserica (s. l.) abnormoides sp. n.

Taxon classificationAnimaliaColeopteraScarabaeidae

http://zoobank.org/5745F0AC-09CA-483C-9D54-B7CA159A0B18

Figs 6A–D , 8

#### Type material examined.

Holotype: ♂ “N. Vietnam (Tonkin) Tamdao 12.-24.5.1989 Pacholátko Leg./ Coll. P. Pacholátko Invt. No./ VS 45” (CPPB). Paratype: 1 ♂ “Mt. Wuyanling, Zhejiang, 3.VIII.2007, light trap, leg. Zhu Weibing” (NKUT).

#### Description.

Body length: 12.7 mm, length of elytra: 9.2 mm, width: 7.6 mm. Body oblong, dark brown, antennal club yellowish brown, anterior labroclypeus shiny, dorsal surface dull, sparsely setose.

Labroclypeus subtrapezoidal, distinctly wider than long, widest at base, lateral margins moderately convex and convergent anteriorly, anterior angles strongly rounded, anterior margin weakly sinuate medially, margins moderately reflexed; surface nearly flat and shiny, basis with dull toment, punctation dense, anteriorly more sparse, behind the anterior margin with coarse punctures each bearing a long erect seta; frontoclypeal suture indistinctly incised, flat and distinctly curved medially; smooth area anterior to eye approximately 1.5 times as wide as long; ocular canthus moderately long (length = 1/3 of ocular diameter) and slender, glabrous, with a long terminal seta. Frons dull, with fine and sparse punctures, beside the eyes with a few erect setae. Eyes small, ratio diameter/interocular width: 0.52. Antenna with ten antennomeres, club with six antennomeres, straight, only slightly longer than the remaining antennomeres combined; antennomere 5 subequal to two thirds of length of club, antennomere 4 slightly transverse, antennomere 3 half as long as pedicellus. Mentum elevated and slightly flattened anteriorly. Labrum distinctly produced medially, with a moderate median sinuation.

Pronotum moderately transverse, subtrapezoidal, widest at base, lateral margins evenly convex, in the basal half only weakly convergent, strongly convergent in anterior half, anterior angles sharp and distinctly produced, posterior angles blunt, slightly rounded at the tip; anterior margin nearly straight, with a distinct and complete marginal line; surface densely and finely punctate with minute setae in punctures; setae of anterior and lateral border sparse; hypomeron basally distinctly carinate, but carina only weakly produced. Scutellum wide and moderately long, triangular with nearly straight sides, apex slightly rounded, with fine, moderately dense punctures, with only minute setae.

Elytra oblong, apex slightly truncate, widest shortly behind the middle, striae weakly impressed, finely and moderately densely punctate, odd intervals slightly convex with punctures concentrated along the striae, others evenly punctate and nearly flat, odd intervals with white scale-like, adpressed setae, otherwise only with very minute setae in punctures; epipleural edge fine, very narrow behind the middle, ending at the blunt external apical angle of elytra, epipleura only sparsely setose, apical border chitinous, with only a very fine fringe of microtrichomes (visible at 100× magnification).

Ventral surface dull, coarsely and densely punctate, metasternum sparsely covered with setae on the disc, glabrous on sides; metacoxa glabrous, with a few short setae laterally, posterior margin weakly convex; abdominal sternites finely and unevenly densely punctuate, nearly glabrous, with a transverse row of coarse punctures, each bearing a robust short seta. Mesosternum between mesocoxae half as wide as slender mesofemur. Ratio of length of metepisternum/metacoxa: 1/1.9. Pygidium weakly convex and dull, coarsely and densely punctate, without smooth midline, with a few semi-erect setae basally on sides, at apex with short, fine, very dense setae.

Legs slender; femora with two longitudinal rows of setae, finely and sparsely punctate between the rows; metafemur dull, anterior margin acute, behind anterior edge without serrated line, setae of anterior longitudinal row nearly completely lacking, posterior margin in apical half ventrally smooth and slightly widened, posterior margin dorsally distinctly serrated, on its basal portion with a few short setae. Metatibia slender and moderately long, widest at apex, ratio of width/length: 1/3.3, sharply carinate dorsally, with two groups of spines, basal group just before the middle, apical group at three quarters of metatibial length, basally with a few robust but single setae; lateral face longitudinally convex, very finely, superficially and sparsely punctate, subdorsal longitudinal carina on lateral face present on about two third of metatibial length; ventral edge finely serrated, with four robust equidistant setae; medial face smooth, apex moderately concavely sinuate interiorly near tarsal articulation. Tarsomeres ventrally with sparse, short setae, laterally not carinate, protarsomeres smooth, meso- and metatarsomeres with a few very fine punctures; metatarsomeres ventrally glabrous, with a strongly serrated ridge ventrally and a sharp subventral carina immediately beside it, first metatarsomere slightly longer than following two tarsomeres combined and slightly longer than dorsal tibial spur. Protibia long, bidentate; anterior claws symmetrical, basal tooth of inner claw sharply truncate at apex.

**Figure 6.**
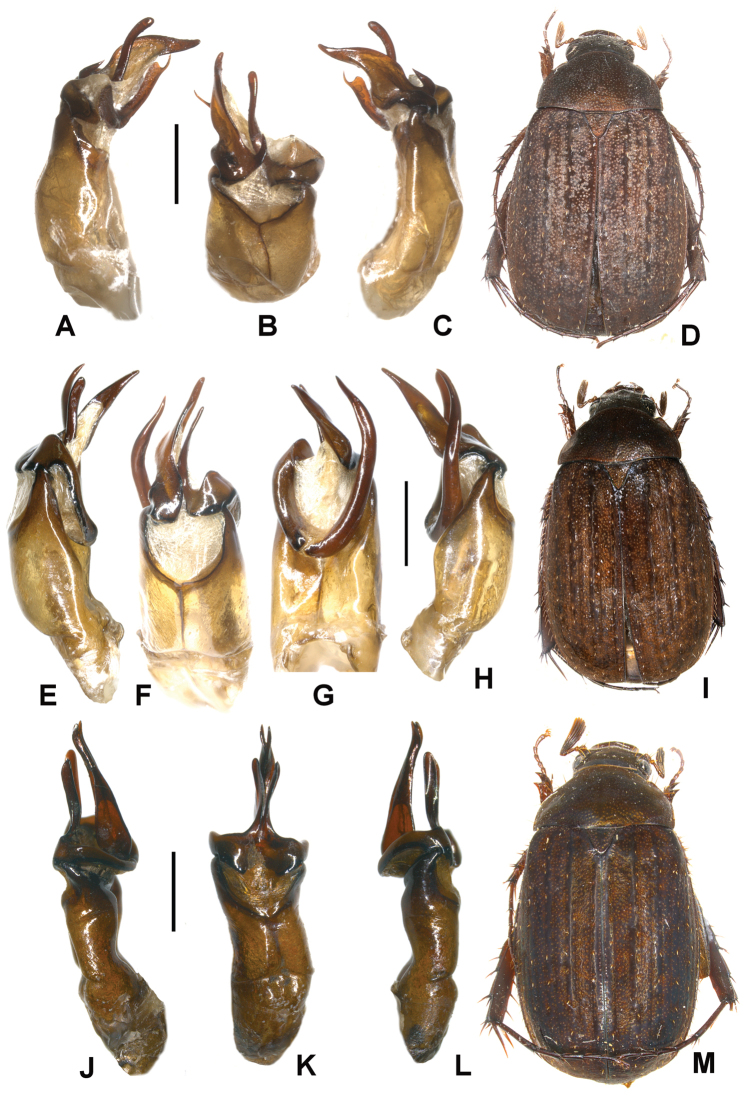
**A–D** *Neoserica abnormoides* sp. n. (holotype) **E–I** *Neoserica cardamomensis* sp. n. (holotype) **J–M** *Neoserica namthaensis* sp. n. (holotype) **A, E, J** Aedeagus, left side lateral view **C, H, L** Aedeagus, right side lateral view **B, F, K** parameres, dorsal view **G** aedeagus, ventral view **D, I, M** Habitus. Scale: 1 mm. Habitus not to scale.

Aedeagus: Fig. 6A–C.

#### Diagnosis.

*Neoserica abnormoides* is in shape of aedeagus very similar to the next species, *Neoserica cardamomensis*. It differs from this species by a small basal hook present at right paramere, and the ventral lobe of the left paramere being distinctly shorter and less strongly curved; the antennal club is composed in male only of six instead of seven antennomeres (as in *Neoserica cardamomensis*).

#### Variation.

Body length: 12.5–12.7 mm, length of elytra: 9.0–9.2 mm, width: 7.5–7.6 mm.

#### Etymology.

The species is named with reference to the externally similar *Neoserica abnormis*.

### Neoserica (s. l.) cardamomensis sp. n.

Taxon classificationAnimaliaColeopteraScarabaeidae

http://zoobank.org/8275FD66-7C1B-40CB-96BF-EA6C3F531FB3

Figs 6E–I , 9

#### Type material examined.

Holotype: ♂ “Cambodia Cardamom Mts., Tumpor area, 12°22'N, 103°02'E, 1250m, Submontane forest, 27.ii-5.iii.2000, leg. M. Nuss” (SMTD). Paratypes: **Cambodia:** 1 ♂ Cambodia Cardamom Mts., Tumpor area, 12°22'N, 103°02'E, 1250m, Submontane forest, 27.ii-5.iii.2000, leg. M. Nuss” (ZFMK). **Thailand:** 1 ♂ “N-Thailand V.1990 Doi Inthanon lg. Malicky” (ZSMC), 1 ♂ “Thai 24–29.IV.1993 Doi Suithep Pacholátko & Dembicky leg./ TS73” (CPPB), 1 ♂ “Thai, 17–24.V.1991 Chiang Dao 1000m 19°25'N, 98°52'E Vit Kuban leg./ Thailand ‘91 “Thanon Thong Cha” D. Král & V. Kubáň” (ZFMK). **Laos:** 1 ♂ “Laos-NE Hua Phan prov., 20°12'N, 104°01'E, Phu Phan Mt., 1500–1900m, 17.5.–3.6.2007, leg. Vit Kuban” (ZFMK), 4 ♂♂ “Laos-NE, Houa Phan prov., 20°13'09–19"N, 103°59'54"-104°00'03"E, 1480–1510m Phou Pane Mt., 22.4.–14.5.2008 Vit Kubáň leg. (NMPC, ZFMK), 2 ♂♂ “Laos-NE, Houa Phan prov., 20°13'09–19"N, 103°59'54"-104°00'03"E, 1480–1510m Phou Pane Mt., 22.IV.–14.V.2008 Vit Kuban leg. (ZFMK), 1 ♂ “Laos-N, Louang Namtha circ., 04.05.1996 I. Pljushtch lg.” (ZFMK), 1 ♂ “Laos-NE, Houa Phan prov., ~20°12–13.5'N, 103°59.5'–104°01'E, Ban Sauei - Phou Pane Mt., 10.–16.vi.2009, 1340–1870 m, M. Brancucci & local collectors leg./ NHMB Basel, NMPC Prague Laos 2009 Expedition: M. Brancucci, M. Geiser, Z. Kraus, D. Hauck, V. Kubáň” (NHMB), 2 ♂♂ “Laos-NE, Houa Phan prov., ~20°12–13.5'N, 103°59.5'–104°01'E, Ban Sauei - Phou Pane Mt., 1340–1870 m, 15.iv-15.v.2008, Lao collectors leg.” (NHMB), 1 ♂ “Laos-NE, Houa Phan prov., 20°13'09–19"N, 103°59'54"–104°00'03"E, 1480–1550m Phou Pane Mt., 1.-16.vi.2009, Zdenek Kraus leg./ NHMB Basel, NMPC Prague Laos 2009 Expedition: M. Brancucci, M. Geiser, Z. Kraus, D. Hauck, V. Kubáň” (NHMB), 1 ♂ “Laos-NE, Houa Phan prov., 20°11–13'N, 103°59'-104°01'E, Ban Sauei - Phou Pane Mt., 9.–17.vi.2009, 1300–1900 m, Michael Geiser leg. / NHMB Basel, NMPC Prague Laos 2009 Expedition: M. Brancucci, M. Geiser, Z. Kraus, D. Hauck, V. Kubáň” (NHMB), 2 ♂♂ “Laos-NE, Xieng Khouang prov., 19°38.20'N, 103°20.20'E, Phonsavan (30 km NE); Phou Sane Mt., 1420 m, 10.–30.v.2009, D. Hauck leg. / NHMB Basel, NMPC Prague Laos 2009 Expedition: M. Brancucci, M. Geiser, Z. Kraus, D. Hauck, V. Kubáň” (NHMB), 1 ♂ “Laos-NE, Houa Phan prov., 20°13'09–19"N, 103°59'54"–104°00'03"E, 1480–1550m Phou Pane Mt., 9.-16.vi.2009, David Hauck leg./ NHMB Basel, NMPC Prague Laos 2009 Expedition: M. Brancucci, M. Geiser, Z. Kraus, D. Hauck, V. Kubáň” (NHMB), 2 ♂♂ “Laos-NE, Xieng Khouang prov., 19°38.20'N, 103°20.20'E, Phonsavan (30 km NE): Phou Sane Mt., 1420 m, 10.-30.v.2009, Z. Kraus leg. / NHMB Basel, NMPC Prague Laos 2009 Expedition: M. Brancucci, M. Geiser, Z. Kraus, D. Hauck, V. Kubáň” (NHMB), 3 ♂♂ “Laos-NE, Xieng Khouang prov., 19°37–38'N, 103°20'E, 30 km NE Phonsavan: Ban Na Lam - Phou Sane Mt., 1300–1500 m, 10.-30.v.2009, M. Geiser leg. / NHMB Basel, NMPC Prague Laos 2009 Expedition: M. Brancucci, M. Geiser, Z. Kraus, D. Hauck, V. Kubáň” (NHMB), 12 ♂♂, 2 ♀♀ “Laos, Attapeau prov.; Annam Highlands Mts Dong Amphan; NBCA, ca. 1160 m Nong Fa (crater lake) env., 15°05.9'N, 107°25.6'E St. Jakl lgt, 30.4.–6.5.2010” (ZFMK), 9 ♂♂, 4 ♀♀ “Laos, Attapeau prov.; Annam Highlands Mts. Dong Amphan NBCA, ca. 1160 m Nong Fa (crater lake) env., 15°05.9'N, 107°25.6'E Jiří Hájek leg. 30.iv.-6.v.2010” (NMPC). **China:** 3 ♂♂ “Mengla, Yunan, 21.IV.1982, leg. Jiang Shengqiao” (IZAS), 1 ♂, 2 ♀♀ “Caiyanghe, Pu'er, Yunnan, 28.VII.2007, leg. Ren Guodong, Hou Wenjun, Li Yalin”(HBUM), 1 ♂ “Mengla, Xishuangbanna, Yunnan, 4.IV.1982, leg. Wang Sumei, Zhou Jingruo” (NUYS), 1 ♂ “Mengla, Yunnan, 20.IV.1982, 670m, light trap, leg. Yu Peiyu” (IZAS).

#### Description.

Body length: 12.0 mm, length of elytra: 9.6 mm, width: 7.6 mm. Body oblong, dark brown, antennal club yellowish brown, anterior labroclypeus shiny, dorsal surface dull, opaque toment on elytra and pronotum less thick, with a light trace of shine, sparsely setose.

Labroclypeus slightly subtrapezoidal, distinctly wider than long, widest at base, lateral margins moderately convex and moderately convergent anteriorly, anterior angles strongly rounded, anterior margin weakly sinuate medially, margins moderately reflexed; surface convexly elevated at middle and shiny, basis with dull toment, punctation dense, anteriorly more sparse, behind the anterior margin with coarse punctures each bearing a long erect seta; frontoclypeal suture distinctly incised, flat and distinctly curved medially; smooth area anterior to eye approximately 1.5 times as wide as long; ocular canthus moderately wide and long (length = 1/3 of ocular diameter), glabrous, with a fine terminal seta. Frons dull, with fine and dense punctures, beside the eyes a with a few erect setae. Eyes small, ratio diameter/interocular width: 0.48. Antenna with ten antennomeres, club with seven antennomeres, straight, 1.2 times as long as remaining antennomeres combined; antennomere 5 subequal to length of club, antennomere 4 more than half as long as the club, antennomere 3 half as long as pedicellus. Mentum elevated and slightly flattened anteriorly. Labrum distinctly produced medially, with a moderate median sinuation.

Pronotum moderately transverse, subtrapezoidal, widest at base, lateral margins evenly convex and convergent anteriorly, anterior angles sharp and distinctly produced, posterior angles blunt, strongly rounded at the tip; anterior margin nearly straight, with a fine and complete marginal line; surface densely and finely punctate with minute setae in punctures; setae of lateral border sparse; hypomeron basally distinctly carinate, but carina only weakly produced. Scutellum long, triangular with nearly straight sides, apex slightly rounded, with fine, dense punctures, impunctate along the middle, with only minute setae.

Elytra oblong, widest shortly behind the middle, striae weakly impressed, finely and moderately densely punctate, odd intervals distinctly convex with punctures concentrated along the striae, others evenly punctate and nearly flat, odd intervals with white scale-like, adpressed setae, otherwise only with very minute setae in punctures; epipleural edge fine, very narrow behind the middle, ending at the moderately rounded external apical angle of elytra, epipleura densely setose, apical border chitinous, with only a very fine fringe of microtrichomes (visible at 100× magnification).

Ventral surface dull, coarsely and densely punctate, metasternum sparsely covered with setae on the disc, glabrous on sides; metacoxa glabrous, with a few short setae laterally, posterior margin weakly convex; abdominal sternites finely and unevenly densely punctuate, nearly glabrous, with a transverse row of coarse punctures, each bearing a robust short seta. Mesosternum between mesocoxae half as wide as slender mesofemur. Ratio of length of metepisternum/metacoxa: 1/1.77. Pygidium moderately convex and dull, densely punctate, fine punctures mixed with coarser ones, without smooth midline, with a few setae beside apical margin.

Legs slender; femora with two longitudinal rows of setae, finely and sparsely punctate between the rows; metafemur dull, anterior margin acute, behind anterior edge without serrated line, setae of anterior longitudinal row nearly completely lacking, posterior margin in apical half ventrally smooth and slightly widened, posterior margin dorsally distinctly serrated, on its basal portion with a few short setae. Metatibia slender and long, widest at apex, ratio of width/length: 1/3.7, sharply carinate dorsally, with two groups of spines, basal group just before the middle, apical group at three quarters of metatibial length, basally with a few robust but single setae; lateral face longitudinally convex, very finely, superficially and sparsely punctate, subdorsal longitudinal carina on lateral face present on about two third of metatibial length; ventral edge finely serrated, with three robust equidistant setae; medial face smooth, apex moderately concavely sinuate interiorly near tarsal articulation. Tarsomeres ventrally with sparse, short setae, laterally not carinate, protarsomeres smooth, meso- and metatarsomeres with a few very fine punctures; metatarsomeres ventrally glabrous, with a strongly serrated ridge ventrally and a sharp subventral carina immediately beside it, first metatarsomere as long as following two tarsomeres combined and slightly longer than dorsal tibial spur. Protibia long, bidentate; anterior claws symmetrical, basal tooth of inner claw sharply truncate at apex.

Aedeagus: Fig. 6E–H.

#### Diagnosis.

*Neoserica cardamomensis* sp. n. differs from all other species of the *Neoserica abnormis* group by an antennal club of males composed of seven antennomeres.

#### Variation.

Body length: 11.4–14.1 mm, length of elytra: 8.5–10.5 mm, width: 6.1–7.6 mm. Female: Antennal club composed of five antennomeres, club as long as remaining antennomeres combined, 6^th^ antennomere subequal three quarters of club length, 7^th^ antennomere slightly transverse; disc of pygidium less convex than in male, only at apex strongly convex.

#### Etymology.

The new species is named after the Cardamom Mountains (Cambodia) where its type locality is situated.

### Neoserica (s. l.) namthaensis sp. n.

Taxon classificationAnimaliaColeopteraScarabaeidae

http://zoobank.org/1782971E-9F1C-4855-AE23-71E9F5A6BCEE

Figs 6J–M , 8

#### Type material examined.

Holotype: ♂ “Laos, 21°09'N, 101°19'E, Louangnamtha pr. Namtha-> MuangSing, 5.–31.v.1997, 900-1200m Vit Kubáň leg./ LS19” (CPPB).

#### Description.

Body length: 11.8 mm, length of elytra: 8.2 mm, width: 6.5 mm. Body oblong, dark brown, antennal club yellowish brown, anterior labroclypeus shiny, dorsal surface dull, opaque toment on elytra and pronotum less thick, with a light trace of shine, sparsely setose.

Labroclypeus subrectangular, distinctly wider than long, widest at base, lateral margins moderately convex and very little convergent anteriorly, anterior angles strongly rounded, anterior margin weakly sinuate medially, margins moderately reflexed; surface convexly elevated at middle and shiny, basis with dull toment, punctation moderately dense, anteriorly more sparse, behind the anterior margin with coarse punctures each bearing a long erect seta; frontoclypeal suture distinctly incised, flat and distinctly curved medially; smooth area anterior to eye approximately 1.5 times as wide as long; ocular canthus moderately wide and long (length = 1/3 of ocular diameter), glabrous, with a fine terminal seta. Frons dull, with fine and dense punctures, beside the eyes a with a few erect setae, otherwise with minute setae only. Eyes small, ratio diameter/interocular width: 0.5. Antenna with ten antennomeres, club with seven antennomeres, straight, 1.2 times as long as remaining antennomeres combined; antennomere 5 subequal to length of club, antennomere 4 0.7 times as long as the club, antennomere 3 half as long as pedicellus. Mentum elevated and slightly flattened anteriorly. Labrum distinctly produced medially, with a moderate median sinuation.

Pronotum moderately transverse, subtrapezoidal, widest at base, lateral margins weakly convex and in basal half weakly convergent anteriorly, at middle more convex and in anterior half weakly convex and strongly convergent, anterior angles moderately sharp and distinctly produced, posterior angles blunt, moderately rounded at the tip; anterior margin nearly straight, with a fine and complete marginal line; surface densely and finely punctate with minute setae in punctures; setae of lateral border sparse; hypomeron basally distinctly carinate, but carina only weakly produced. Scutellum long, triangular with nearly straight sides, apex slightly rounded, with fine, dense punctures, impunctate along the middle, with only minute setae.

Elytra oblong, widest shortly behind the middle, striae weakly impressed, finely and moderately densely punctate, odd intervals distinctly convex with punctures concentrated along the striae, others evenly punctate and nearly flat, odd intervals with white scale-like, adpressed setae, otherwise only with very minute setae in punctures; epipleural edge fine, very narrow behind the middle, ending at the moderately rounded external apical angle of elytra, epipleura densely setose, apical border chitinous, with only a very fine fringe of microtrichomes (visible at 100× magnification).

Ventral surface dull, coarsely and densely punctate, metasternum sparsely covered with setae on the disc, glabrous on sides; metacoxa glabrous, with a few short setae laterally, posterior margin weakly convex; abdominal sternites finely and unevenly densely punctuate, nearly glabrous, with a transverse row of coarse punctures, each bearing a robust short seta. Mesosternum between mesocoxae half as wide as slender mesofemur. Ratio of length of metepisternum/metacoxa: 1/1.8. Pygidium moderately convex and dull, densely punctate, fine punctures mixed with coarser ones, with a narrow smooth midline, with a few longer setae on disc and beside the apical margin.

Legs moderately slender; femora with two longitudinal rows of setae, finely and sparsely punctate between the rows; metafemur dull, anterior margin acute, behind anterior edge without serrated line, setae of anterior longitudinal row nearly completely lacking, posterior margin in apical half ventrally smooth and slightly widened, posterior margin dorsally distinctly serrated, on its basal portion with a few short setae. Metatibia moderately slender and long, widest at apex, ratio of width/length: 1/3.2, sharply carinate dorsally, with two groups of spines, basal group just before the middle, apical group at three quarters of metatibial length, basally with a few robust but single setae; lateral face longitudinally convex, very finely, superficially and sparsely punctate, subdorsal longitudinal carina on lateral face present on about two third of metatibial length; ventral edge finely serrated, with three robust equidistant setae; medial face smooth, apex moderately concavely sinuate interiorly near tarsal articulation. Tarsomeres ventrally with sparse, short setae, laterally not carinate, protarsomeres smooth, meso- and metatarsomeres with a few very fine punctures; metatarsomeres ventrally glabrous, with a strongly serrated ridge ventrally and a sharp subventral carina immediately beside it, first metatarsomere as long as following two tarsomeres combined and slightly longer than dorsal tibial spur. Protibia long, bidentate; anterior claws symmetrical, basal tooth of inner claw sharply truncate at apex.

Aedeagus: Fig. 6J–L.

#### Diagnosis.

*Neoserica namthaensis* sp. n. differs from all other species (except the previous one) of the *Neoserica abnormis* group by having an antennal club in male composed of seven antennomeres. From *Neoserica cardamomensis* it can be distinguished by the nearly symmetrical parameres.

#### Etymology.

The new species is named according to its type locality in the environment of Namtha (Laos).

### Neoserica (s. l.) bairailingshanica sp. n.

Taxon classificationAnimaliaColeopteraScarabaeidae

http://zoobank.org/6EB277CE-503A-46DE-9539-FCE098EDB267

Figs 7A–D , 9

#### Type material examined.

Holotype: ♂ “China: N-Yunnan, Baiyungshan (Bai Railing Mts.) 2400m, Yong Ren, VII-2003 leg. Ying et al.” (ZFMK).

#### Description.

Body length: 11.7 mm, length of elytra: 8.1 mm, width: 6.6 mm. Body oblong, dark brown, antennal club yellowish brown, anterior labroclypeus shiny, dorsal surface dull, nearly glabrous.

Labroclypeus subrectangular, little wider than long, widest at base, lateral margins straight and weakly convergent anteriorly, anterior angles moderately rounded, anterior margin distinctly sinuate medially, margins strongly reflexed; surface flat and shiny, basis with dull toment, punctation dense, behind the anterior margin with coarser punctures each bearing a long erect seta; frontoclypeal suture indistinctly incised, flat and distinctly curved medially; smooth area anterior to eye approximately twice as wide as long; ocular canthus moderately long (length = 1/3 of ocular diameter) and slender, glabrous, with a few minute and superficial punctures, with a long terminal seta. Frons dull, in posterior half weakly shiny, with fine and sparse punctures, beside the eyes and behind the frontoclypeal suture with a few erect setae. Eyes large, ratio diameter/interocular width: 0.76. Antenna with ten antennomeres, club with six antennomeres, moderately reflexed, 1.5 times as long as remaining antennomeres combined; antennomere 5 subequal to half of length of club, antennomere 4 moderately transverse, antennomere 3 half as long as pedicellus. Mentum elevated and slightly flattened anteriorly. Labrum weakly produced medially, with a moderate median sinuation.

Pronotum transverse, subrectangular, widest just before base, lateral margins in basal half straight and nearly subparallel, slightly narrowed towards the base, evenly convex and strongly convergent in anterior half, anterior angles moderately sharp and moderately produced, posterior angles blunt, slightly rounded at the tip; anterior margin strongly convexly produced medially, with a medially incomplete marginal line; surface densely and finely punctate, with minute setae in punctures; setae of lateral border fine and sparse; hypomeron basally distinctly carinate, but carina not produced. Scutellum moderately wide and long, with fine, moderately dense punctures, smooth on basal midline, with only minute setae.

Elytra oblong, widest in posterior third, striae weakly impressed, finely and moderately densely punctate, intervals weakly convex with punctures concentrated along the striae, odd intervals with fine, adpressed setae, otherwise only with very minute setae in punctures; epipleural edge fine, ending at the blunt external apical angle of elytra, epipleura densely setose, apical border narrowly membraneous, with a fine fringe of microtrichomes (visible at 100× magnification).

Ventral surface dull, coarsely and densely punctate, metasternum glabrous; metacoxa glabrous, with a few short setae laterally, posterior margin straight; abdominal sternites finely and unevenly and not densely punctuate, nearly glabrous, with a transverse row of coarse punctures, each bearing a robust short seta. Mesosternum between mesocoxae half as wide as slender mesofemur. Ratio of length of metepisternum/metacoxa: 1/1.4. Pygidium weakly convex and dull, finely and densely punctate, without a smooth midline, with a few longer setae between the minute setae.

Legs slender; femora with two longitudinal rows of setae, finely and sparsely punctate between the rows; metafemur dull, anterior margin acute, behind anterior edge without serrated line, punctures and setae of anterior longitudinal row completely reduced, posterior margin in apical half ventrally smooth and not widened and smooth in apical quarter, posterior margin dorsally distinctly serrated, on its basal portion with a few short setae. Metatibia slender and long, widest at apex, ratio of width/length: 1/5.1, sharply carinate dorsally, with two groups of spines, basal group just before the middle, apical group at three quarters of metatibial length, basally with a few robust but single setae; lateral face longitudinally convex, very finely, superficially and sparsely punctate, subdorsal longitudinal carina on lateral face present on about two third of metatibial length, but not very distinct; ventral edge finely serrated, with three robust setae of which the two distal ones are widely separated; medial face smooth, apex moderately distinctly concavely sinuate interiorly near tarsal articulation. Tarsomeres ventrally with sparse, short setae, laterally not carinate, protarsomeres smooth, meso- and metatarsomeres with dense, fine punctures; metatarsomeres with a strongly serrated ridge ventrally and a sharp subventral carina immediately beside it, first metatarsomere as long as following two tarsomeres combined and slightly longer than dorsal tibial spur. Protibia long, bidentate; anterior claws symmetrical, basal tooth of inner claw sharply truncate at apex.

**Figure 7.**
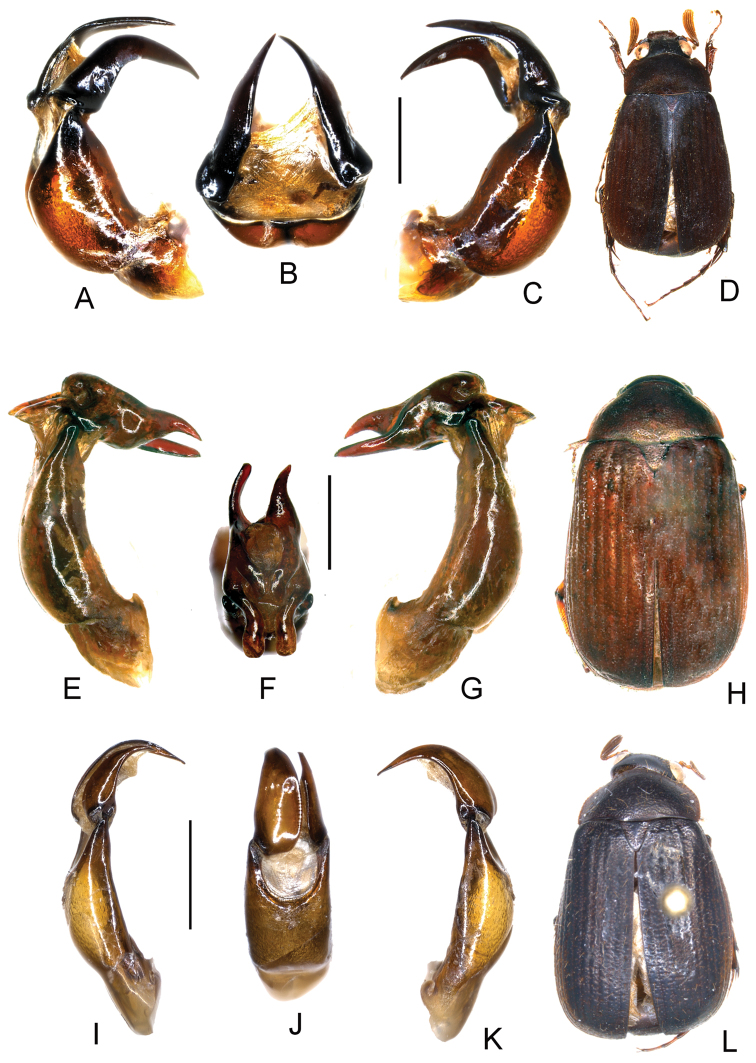
**A–D** *Neoserica bairailingshanica* sp. n. (holotype) **E–H** *Neoserica huangi* sp. n. (holotype) **I–L** *natmatoungensis* sp. n. (holotype) **A, E, I** Aedeagus, left side lateral view **C, G, K** Aedeagus, right side lateral view **B, F, J** parameres, dorsal view **D, H, L** Habitus. Scale: 1 mm. Habitus not to scale.

Aedeagus: Fig. 7A–C. Female unknown.

#### Diagnosis.

The new species differs from all other species of the *Neoserica abnormis* group by the convexly produced anterior margin of pronotum and the lacking scale-like setae on elytra. From *Neoserica trifida* it differs by the smooth ventral posterior margin of metafemur, by the slightly shorter antennal club of male, and of course by shape of parameres being simple and not subdivided into separate lobes.

#### Etymology.

The new species is named according to its occurrence in the Bai Railing Shan.

### Neoserica (s. l.) huangi sp. n.

Taxon classificationAnimaliaColeopteraScarabaeidae

http://zoobank.org/9E196146-26B7-45C5-ABA7-24BBABCAC294

Figs 7E–H , 9

#### Type material examined.

Holotype ♂ [China] “Dongqiong, Chayu, Xizang, 16.VII.1973, 1850–2500m, leg. Huang Fusheng” (IZAS).

#### Description.

Body length: 11.8 mm, length of elytra: 8.8 mm, width: 6.8 mm. Body oblong, dark brown, antennal club brown, anterior labroclypeus shiny, dorsal surface dull, densely covered with minute erect setae.

Labroclypeus subtrapezoidal, distinctly wider than long, widest at base, lateral margins moderately convex and convergent anteriorly, anterior angles moderately rounded, anterior margin weakly sinuate medially, margins moderately reflexed; surface nearly flat and shiny, basis with dull toment, punctation moderately dense and superficial, punctures each bearing a long erect seta; frontoclypeal suture indistinctly incised, flat and distinctly curved medially; smooth area anterior to eye nearly as wide as long; ocular canthus short (length =1/4 of ocular diameter) and wide, densely and minutely setose. Frons dull, with fine and dense but superficial punctures each bearing a short to minute seta, beside eyes and behind the frontoclypeal suture with a few longer erect setae. Eyes small, ratio diameter/interocular width: 0.46. Antenna with ten antennomeres, club with six antennomeres, straight, as long as remaining antennomeres combined; antennomere 5 two thirds as long as the club, antennomere 4 slightly transverse, antennomere 3 half as long as pedicellus. Mentum elevated and slightly flattened anteriorly. Labrum distinctly produced medially, with a moderate median sinuation.

Pronotum moderately transverse, subtrapezoidal, widest at base, lateral margins evenly convex and strongly convergent anteriorly, anterior angles sharp and distinctly produced, posterior angles blunt, slightly rounded at the tip; anterior margin weakly convex medially, marginal line widely interrupted medially; surface densely and finely punctate, with short to minute erect setae in punctures; setae of anterior and lateral border absent; hypomeron basally distinctly carinate, but carina only weakly produced. Scutellum moderately long, subtriangular, with fine, moderately dense punctures, impunctate on basal midline, with minute erect setae in punctures.

Elytra oblong, widest at posterior third, striae finely impressed, finely and moderately densely punctate, intervals nearly flat, with moderately dense evenly spaced, fine punctures each bearing a short fine erect setae, odd intervals with a few fine longer setae; epipleural edge fine, ending at the blunt external apical angle of elytra, epipleura sparsely setose, apical border chitinous, with only a very fine fringe of microtrichomes (visible at 100× magnification).

Ventral surface dull, coarsely and densely punctate, densely covered with short setae; metacoxa glabrous, with a very few short setae laterally, posterior margin weakly convex; abdominal sternites finely and unevenly densely punctuate, densely and finely setose, with a transverse row of coarse punctures, each bearing a robust short seta. Mesosternum between mesocoxae half as wide as slender mesofemur. Ratio of length of metepisternum/metacoxa: 1/1.43. Pygidium weakly convex and dull, coarsely and densely punctate, without smooth midline, densely and finely setose, setae on apical half longer.

Legs slender; femora with two longitudinal rows of setae, finely and sparsely punctate between the rows; metafemur dull, anterior margin acute, behind anterior edge without serrated line, anterior longitudinal row complete, posterior margin in apical half ventrally smooth and slightly widened, posterior margin dorsally distinctly serrated, on its basal portion with a few short setae. Metatibia slender and long, widest at apex, ratio of width/length: 1/3.9, sharply carinate dorsally, with two groups of spines, basal group just before the middle, apical group at three quarters of metatibial length, basally with a few robust but single setae; lateral face longitudinally convex, impunctate, subdorsal longitudinal carina on lateral face present on about two third of metatibial length; ventral edge finely serrated, with three robust equidistant setae; medial face smooth, apex moderately concavely sinuate interiorly near tarsal articulation. Tarsomeres ventrally with sparse, short setae, laterally not carinate, protarsomeres smooth, meso- and metatarsomeres with a few very fine punctures; metatarsomeres ventrally glabrous, with a strongly serrated ridge ventrally and a sharp subventral carina immediately beside it, first metatarsomere slightly longer than following two tarsomeres combined and slightly longer than dorsal tibial spur. Protibia long, bidentate; anterior claws symmetrical, basal tooth of inner claw sharply truncate at apex.

Aedeagus: Fig. 7E–G. Female unknown.

#### Diagnosis.

*Neoserica huangi* differs from all other species of the *Neoserica abnormis* group in the minute to short, dense erect pilosity of the body, as well as the nearly subsymmetrical parameres (basal half).

#### Etymology.

The new species is named after its collector, Huang Fusheng.

### Neoserica (s. l.) natmatoungensis sp. n.

Taxon classificationAnimaliaColeopteraScarabaeidae

http://zoobank.org/D790AEAF-BEAA-47FC-903E-F167983D4896

Figs 7I–L , 9

#### Type material examined.

Holotype ♂ “Myanmar (Burma), Province Chin/ Chin Hills Umg. Kanpetlet Natmatoung N.P. (NF), 23.VI.2008 leg. Michael Langer/ E 093°57'N, 21°13' H= ca. 1500m” (ZFMK). Paratypes: 3 ♂♂ “Myanmar (Burma), Province Chin/ Chin Hills Umg. Kanpetlet Natmatoung N.P. (NF), 23.VI.2008 leg. Michael Langer/ E 093°57'N, 21°13' H= ca. 1500m” (ZFMK).

#### Description.

Body length: 11 mm, length of elytra: 7.6 mm, width: 5.2 mm. Body oblong, dark brown, ventral surface and antennal yellowish brown, anterior labroclypeus shiny, dorsal surface moderately shiny, frons dull, with numerous long, yellow, semi-erect setae.

Labroclypeus subtrapezoidal, little wider than long, widest at base, lateral margins weakly convex and convergent anteriorly, anterior angles moderately rounded, anterior margin weakly sinuate medially, margins strongly reflexed; surface flat and shiny, basis without dull toment, finely and densely punctate, with numerous coarser punctures interspersed each bearing a long erect seta; frontoclypeal suture indistinctly incised, flat and distinctly curved medially; smooth area anterior to eye approximately twice as wide as long; ocular canthus long and slender, impunctate and glabrous, with a long terminal seta. Frons dull, finely and densely punctate, with numerous coarser punctures interspersed each bearing a long erect seta. Eyes moderately large, ratio diameter/interocular width: 0.69. Antenna with ten antennomeres, club with five antennomeres, straight, as long as remaining antennomeres combined; antennomere 6 subequal to two thirds of length of club, antennomere 5 transverse. Mentum elevated and slightly flattened anteriorly. Labrum weakly produced medially, with a moderate median sinuation.

Pronotum transverse, subrectangular, widest just before base, lateral margins evenly convex and strongly convergent towards sharp and moderately produced anterior angles, posterior angles blunt, rounded at the tip; anterior margin strongly convexly produced medially, marginal line robust and complete medially; surface densely and finely punctate, with coarse punctures irregularly interspersed each bearing a long semi-erect seta, fine punctures minutely setose; setae of lateral and anterior border dense; hypomeron basally distinctly carinate, but carina not produced. Scutellum moderately wide and short, with fine, dense punctures, widely smooth on basal midline, with only minute setae.

Elytra oblong, widest at middle, striae weakly impressed, finely and moderately densely punctate, intervals weakly convex with fine and moderately dense punctures concentrated along striae, all intervals with sparse semi-erect long setae, otherwise punctures only with very minute setae; epipleural edge fine, ending at the blunt external apical angle of elytra, epipleura densely setose, apical border chitinous, without fringe of microtrichomes (visible at 100× magnification).

Ventral surface shiny, coarsely densely punctate and finely setose; metacoxa glabrous, with a few short setae laterally, posterior margin straight; abdominal sternites finely and densely punctuate, with minute setae in punctures, each sternite with a transverse row of coarse punctures each bearing a robust short seta. Mesosternum between mesocoxae half as wide as slender mesofemur. Ratio of length of metepisternum/metacoxa: 1/1.47. Pygidium weakly convex and moderately shiny, coarsely and densely punctate, on basal portion also with finer punctures interspersed, without a smooth midline, with dense, long setae.

Legs slender; femora with two longitudinal rows of setae, finely and sparsely punctate between the rows; metafemur shiny, anterior margin acute, behind anterior edge without serrated line, punctures and setae of anterior longitudinal row complete, posterior margin in apical half ventrally smooth and neither widened nor serrate in apical quarter, posterior margin dorsally distinctly serrated, on its basal portion with a very few short setae. Metatibia slender and long, widest at apex, ratio of width/length: 1/4.3, sharply carinate dorsally, with two groups of spines, basal group at one third, apical group at three quarters of metatibial length, basally with a few robust but single setae; lateral face longitudinally convex, finely and sparsely punctate, subdorsal longitudinal carina on lateral face present on about two third of metatibial length; ventral edge finely serrated, with three robust setae of which the two distal ones are widely separated; medial face smooth, apex moderately distinctly concavely sinuate interiorly near tarsal articulation. Tarsomeres ventrally with sparse, short setae, laterally not carinate, protarsomeres smooth, meso- and metatarsomeres with a few fine punctures; metatarsomeres with a strongly serrated ridge ventrally and a sharp subventral carina immediately beside it, first metatarsomere as long as following two tarsomeres combined and distinctly longer than dorsal tibial spur. Protibia long, bidentate; anterior claws symmetrical, basal tooth of inner claw sharply truncate at apex.

Aedeagus: Fig. 7I–K. Female unknown.

#### Diagnosis.

The new species differs from all other species of the *Neoserica abnormis* group in the antennal club composed of five antennomeres, while it is entirely different from *Lepidoserica* by the strongly produced anterior angles of pronotum and the unproduced hypomeron.

#### Variation.

Body length: 9.5–11 mm, length of elytra: 7.0–7.6 mm, width: 5.1–5.2 mm.

#### Etymology.

The new species is named after its type locality, the Natmatoung National Park.

### Neoserica (s. l.) ponderosa

Taxon classificationAnimaliaColeopteraScarabaeidae

(Arrow, 1946) comb. n.

Fig. 8

Serica ponderosa Arrow, 1946: 11; Ahrens and Sabatinelli 1996: 209, 239; Ahrens 2012: 315.

#### Type material examined.

Syntypes: 1 ♂ “N. E. Burma R. Malaise B. M. 1945-71/ N. E. Burma Kambaiti 7000 ft. 22/4/1934 R. Malaise/ Co-Type/ Serica ponderosa co-type Arrow“ (BMNH), 1 ♂ “N. E. Burma R. Malaise B. M. 1945-71/ N. E. Burma Kambaiti 7000 ft. 22/6/1934 R. Malaise/ Co-Type/ Serica ponderosa co-type Arrow“ (BMNH), 1 ♂ “N.E. Burma Kambaiti, 2000 m 29/5. 1934 Malaise/ Typus/ Serica ponderosa n.sp. Arrow/ 3136 E91/Naturhistoriska Riksmuseet Stockholm Loan no 559/94” (NHRS), 1 ♂ “N.E. Burma Kambaiti, 7000 ft. 4-8/6. 1934 R. Malaise/ Serica ponderosa co-type Arrow/ ponderosa Arrow/ 3137 E91/Naturhistoriska Riksmuseet Stockholm Loan no 560/94” (NHRS).

#### Remarks.

The aedeagus of a syntype of *Neoserica ponderosa* was illustrated by Ahrens and Sabatinelli (1996) [p. 239]. This species is known so far only from the type locality.

**Figure 8.**
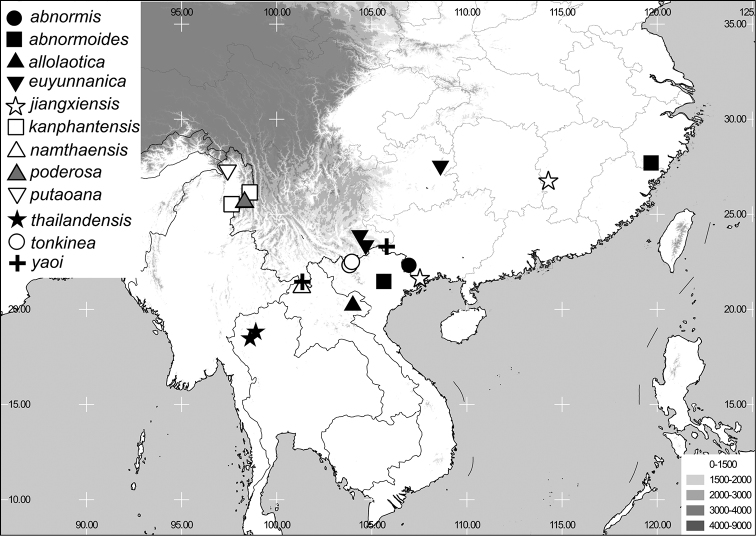
Distribution of the species of the Neoserica (s. l.) abnormis group: *Neoserica abnormis*, *Neoserica abnormoides*, *Neoserica allolaotica*, *Neoserica euyunnanica*, *Neoserica jiangxiensis*, *Neoserica kanphantensis*, *Neoserica namthaensis*, *Neoserica ponderosa*, *Neoserica putaoana*, *Neoserica thailandensis*, *Neoserica tonkinea*, *Neoserica yaoi*.

**Figure 9.**
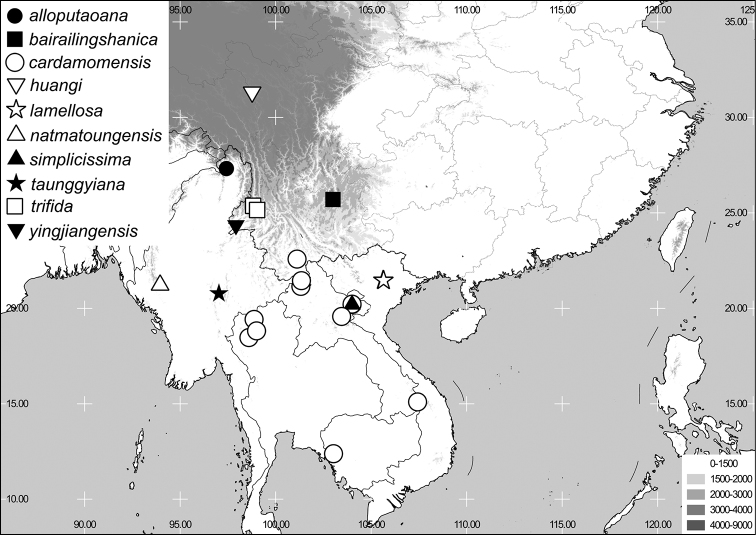
Distribution of the species of the Neoserica (s. l.) abnormis group: *Neoserica alloputaoana*, *Neoserica bairailingshanica*, *Neoserica cardamomensis*, *Neoserica huangi*, *Neoserica lamellosa*, *Neoserica natmatoungensis*, *Neoserica simplicissima*, *Neoserica taunggyiana*, *Neoserica trifida*, *Neoserica yingjiangensis*.

### Uncertain taxonomic status

#### Neoserica (s. l.) inclinata

Taxon classificationAnimaliaColeopteraScarabaeidae

Brenske, 1898

Neoserica inclinata Brenske, 1898: 371 [type locality: Cochinchina].

##### Type material examined.

Lectotype: ♀ “56942/ inclinata Type Brsk/ Conchin Morsb./ inclinata Brsk.* Hinter-Ind.” (ZMHB).

##### Remarks.

The specimen preserved in the ZHMB is a female and not a male as stated by Brenske (1898). Unfortunately, its genitalia are strongly damaged, and therefore an assignment to *Neoserica abnormis* or any of the other newly recognised species is not possible. This species very likely belongs to the *Neoserica abnormis* group as well.

## Discussion

A number of *Neoserica* (s. l.) species that are treated herein resemble *Neoserica abnormis* in external morphology. However, they do not share principal diagnostic features of *Neoserica abnormis* and its closest allies (i.e. elytra with sparse, short, white, scale-like setae and small eyes). These taxa include *Neoserica huangi*, *Neoserica trifida*, *Neoserica yingjiangensis*, *Neoserica bairailingshanica*, and *Neoserica natmatoungensis*. Their systematic position could not be defined here in detail. They would fall in that group of taxa more or less (so far not further defined) widely related with the *Neoserica abnormis* group, but also with *Nepaloserica*, *Chrysoserica*, and *Neoserica barberi* group from India.

The same is valid for Neoserica (s. l.) ponderosa (Arrow) which quite likely does not belong to the *Neoserica abnormis* group. In the morphology-based phylogenetic analysis (Ahrens 2012), it resulted as the sister taxon of the genus *Nepaloserica*. Since the major diagnostic features of *Nepaloserica* (Frey 1965) are lacking in this species (antennal club composed of six antennomeres rather than seven), it seems reasonable to not assign it now to *Nepaloserica*.

And, last but not least, diagnostic features used in the key above to circumscribe the *Neoserica abnormis* group and to classify the species treated here, are not very suitable to support its monophyly. Given also the huge diversity in genital morphology within these species, it could be that the species belong to separate lineages in respect to *Chrysoserica*, *Nepaloserica*, and *Neoserica abnormis*.

Major efforts are needed to obtain DNA samples of these species in order to reconstruct their phylogeny more robustly and to reveal possibly novel traits that may be helpful for the genus systematics.

## Supplementary Material

XML Treatment for Neoserica (s. l.) abnormis

XML Treatment for Neoserica (s. l.) yaoi

XML Treatment for Neoserica (s. l.) allolaotica

XML Treatment for Neoserica (s. l.) tonkinea

XML Treatment for Neoserica (s. l.) taunggyiana

XML Treatment for Neoserica (s. l.) euyunnanica

XML Treatment for Neoserica (s. l.) jiangxiensis

XML Treatment for Neoserica (s. l.) kanphantensis

XML Treatment for Neoserica (s. l.) trifida

XML Treatment for Neoserica (s. l.) yingjiangensis

XML Treatment for Neoserica (s. l.) thailandensis

XML Treatment for Neoserica (s. l.) lamellosa

XML Treatment for Neoserica (s. l.) putaoana

XML Treatment for Neoserica (s. l.) alloputaoana

XML Treatment for Neoserica (s. l.) simplicissima

XML Treatment for Neoserica (s. l.) abnormoides

XML Treatment for Neoserica (s. l.) cardamomensis

XML Treatment for Neoserica (s. l.) namthaensis

XML Treatment for Neoserica (s. l.) bairailingshanica

XML Treatment for Neoserica (s. l.) huangi

XML Treatment for Neoserica (s. l.) natmatoungensis

XML Treatment for Neoserica (s. l.) ponderosa

XML Treatment for Neoserica (s. l.) inclinata
